# Smartphone Keystroke Biomarkers as Predictors of Adverse Neuropsychiatric Sequelae After Trauma in Trauma Survivors: Prospective Observational Cohort Study

**DOI:** 10.2196/73771

**Published:** 2026-06-01

**Authors:** Nicole A Short, Xinming An, Yinyao Ji, Qinghua Li, Thomas C Neylan, Gari D Clifford, Stacey L House, Francesca L Beaudoin, Jennifer S Stevens, Sarah D Linnstaedt, Laura T Germine, John P Haran, Alan B Storrow, Christopher Lewandowski, Paul I Musey Jr, Phyllis L Hendry, Sophia Sheikh, Christopher W Jones, Brittany E Punches, Jose L Pascual, Mark J Seamon, Erica Harris, Claire Pearson, Roland C Merchant, Robert M Domeier, Niels K Rathlev, Brian J O'Neil, Paulina Sergot, Leon D Sanchez, Steven E Bruce, Ronald C Kessler, Karestan C Koenen, Kerry J Ressler, Samuel A McLean

**Affiliations:** 1 Department of Psychology University of Nevada, Las Vegas Las Vegas, NV United States; 2 Department of Anesthesiology University of North Carolina at Chapel Hill Chapel Hill, NC United States; 3 Department of Psychiatry University of California, San Francisco San Francisco, CA United States; 4 Department of Neurology University of California, San Francisco San Francisco, CA United States; 5 Department of Psychiatry and Behavioral Sciences Emory University Atlanta, GA United States; 6 Department of Emergency Medicine Washington University in St. Louis St. Louis, MO United States; 7 Department of Epidemiology Brown University Providence, RI United States; 8 Department of Psychiatry McLean Hospital Belmont, MA United States; 9 Department of Emergency Medicine University of Massachusetts Chan Medical School Worcester, MA United States; 10 Department of Emergency Medicine Vanderbilt University Medical Center Nashville, TN United States; 11 Department of Emergency Medicine Henry Ford Health System Detroit, MI United States; 12 Department of Emergency Medicine Indiana University School of Medicine Indianapolis, IN United States; 13 Department of Emergency Medicine University of Florida Jacksonville, FL United States; 14 Department of Emergency Medicine Cooper Medical School of Rowan University Camden, SC United States; 15 Department of Emergency Medicine The Ohio State University Columbus, OH United States; 16 Department of Surgery University of Pennsylvania Philadelphia, PA United States; 17 Department of Emergency Medicine Einstein Medical Center Philadelphia Philadelphia, PA United States; 18 Department of Emergency Medicine Wayne State University Detroit, MI United States; 19 Department of Emergency Medicine Brigham and Women's Hospital Boston, MA United States; 20 Department of Emergency Medicine Trinity Health Ann Arbor Hospital Ypsilanti, MI United States; 21 Department of Emergency Medicine University of Massachusetts Chan Medical School Springfield, MA United States; 22 Department of Emergency Medicine The University of Texas Houston, TX United States; 23 Department of Psychological Sciences University of Missouri–St. Louis St. Louis, MO United States; 24 Department of Epidemiology Harvard University Boston, MA United States

**Keywords:** trauma, keystroke, posttraumatic stress, pain, somatic symptoms, sleep, mHealth

## Abstract

**Background:**

Adverse posttraumatic neuropsychiatric sequelae are common after trauma. Early identification of individuals at risk for these outcomes could enable the deployment of preventive interventions to survivors at greatest risk. Smartphone keystroke biomarkers show promise in identifying individuals with neuropsychiatric symptoms; however, to our knowledge, no research has examined whether they can be used to identify symptoms in the aftermath of trauma.

**Objective:**

This study evaluates whether passively collected keystroke data from smartphone use in daily life could identify individuals with high symptom levels, as well as worsening or recovery of symptoms, after trauma exposure.

**Methods:**

Data from a diverse cohort of individuals presenting to 27 emergency departments after trauma were analyzed. Inclusion criteria were presenting to the emergency department within 72 hours of trauma, age 18-75, and the ability to speak and read English. Exclusion criteria were solid organ injury, significant hemorrhage, operative intervention, or likely admission for over 72 hours. Participants installed an app that passively collected keystroke data during use of any app on their smartphone, beginning in the emergency department. Participants also completed serial symptom assessments over 8 weeks after trauma exposure.

**Results:**

A total of 3445 patients met study criteria, provided informed consent, and completed assessments in the emergency department. Of these, 1072 (mean age 40, SD 13; 616/1072, 57.46%, women; 565/1072, 52.71% non–Hispanic Black) installed the app on their Android smartphone and completed the 8-week assessment and were therefore included in analyses. Keystroke biomarkers related to typing speed, identified using bivariate linear mixed models controlling for false discovery rates, were associated with elevated pain, reexperiencing, and mental fatigue (absolute values of rs=0.22-0.25, Ps=.02). Separate change-of-operation and scrolling keystroke biomarkers were associated with increased reexperiencing symptoms (r=0.18, *P*=.047) and mental fatigue (rs=0.18-0.19, Ps=.031-.047). Further, changes in specific keystroke biomarkers were associated with worsening or recovery of pain (rs=0.07-0.10, Ps=.02), somatic symptoms (rs=0.02, Ps=.02), mental fatigue (rs=0.02-0.04, Ps=.02), sleep disturbance (absolute rs=0.07-0.09, Ps=.02), reexperiencing (rs=0.02-0.04, Ps=.02), and hyperarousal (rs=0.02-0.04, Ps=.02).

**Conclusions:**

In general, slower typing and scrolling speeds were associated with higher symptom levels, with small to medium effect sizes. Keystroke data passively collected via smartphone use may help identify individuals with significant or changing posttraumatic symptoms. Future research should continue to explore these keystroke biomarkers and whether they can be leveraged to connect vulnerable trauma survivors to appropriate services. Overall, these results add to the literature, indicating that passively collected keystroke data may help identify individuals with neuropsychiatric symptoms or changes and are, to our knowledge, the first to test whether keystroke biomarkers are useful in the aftermath of trauma. This represents a critical period during which preventive interventions could be deployed to reduce the long-term burden of trauma-related sequelae.

## Introduction

### Problem

Approximately 9 in 10 Americans experience at least one traumatic stressor during their lifetimes [1]. Most individuals recover naturally; however, adverse posttraumatic neuropsychiatric symptoms (APNSs) are common and morbid [2]. Common APNS domains include pain and other somatic symptoms; depression and anxiety; posttraumatic stress (PTS) symptoms such as reexperiencing, avoidance, and hyperarousal; as well as sleep disruption and nightmares. APNSs are associated with negative consequences, including emotional distress, functional impairments [3,4], and reduced quality of life [5,6].

The vast majority of individuals experience some level of pain or mental health symptoms, particularly PTS, in the immediate aftermath of trauma exposure [7-9]. For most, these symptoms resolve naturally on their own [7,9], whereas for others they become chronic and debilitating conditions [7]. Certain traumas, such as sexual assault, result in a higher risk for specific APNS [10,11]. Despite this, even after “minor” traumas, such as motor vehicle collision (MVC) without serious injury, a large proportion of survivors develop persistent APNSs. Up to half develop persistent pain [8,12-21], and approximately a quarter develop chronic PTS symptoms [22]. Unfortunately, many trauma survivors do not receive trauma-related services outside of emergency care [23]. This is particularly true for individuals who identify as racially minoritized or have lower levels of education and income [24], who are also at higher risk for trauma exposure [25-27]. However, early treatments for such disorders [28,29] could help avoid decades of suffering for trauma survivors, underscoring the importance of identifying at-risk survivors.

### Review of Relevant Scholarship

Nearly 9 in 10 Americans own a smartphone, up from 4 in 10 just 10 years ago [30]. Apps can passively collect data in daily life, including user keystroke behavior. Keystroke patterns may help identify individuals’ risk for mental and physical health problems, including APNSs. Smartphone keystroke behavior data are particularly appealing because they do not require any additional device (eg, wearable), making them feasible to collect given the ubiquity of smartphone use. Research in this area is in nascent stages but suggests that keystroke behavior data, alone [31-33] or in combination with other smartphone data [34], are useful for identifying increased depressive symptoms and cognitive functioning/impairments [35,36] among individuals with depression and bipolar disorder, as well as among healthy controls and military veterans [37]. Furthermore, keystroke dynamics can identify individuals with fine motor impairments [38]. This area of research has tremendous promise, as smartphone use is ubiquitous and could be leveraged to screen for and monitor APNSs in the aftermath of trauma, and to connect those in need to further assessment or care. Although studies have used other types of passively sensed data to detect PTS-related symptoms [39], no large-scale study, to our knowledge, has evaluated the predictive utility of smartphone keystroke behavior for APNSs after trauma.

### Hypotheses, Aims, and Objectives

Given the potential of keystroke behavior data to identify and predict APNSs, the goal of this study was to use longitudinal keystroke data collected from an app installed on participants’ smartphones after trauma exposure to predict APNS outcomes. Specifically, the aims were to test whether keystroke data collected from smartphone use in daily life in a socioeconomically and racially diverse population of trauma survivors presenting to emergency departments (EDs) after traumatic stress exposure can predict risk for, and recovery from or worsening of, APNSs. Individuals with lower socioeconomic status and those identifying with racially/ethnically minoritized groups are at relatively high risk for trauma exposure [25,27] and may experience difficulties accessing appropriate care after the ED visit [23], making them an important sample for targeting preventive interventions. Research Domain Criteria [40]–defined APNSs (ie, pain, reexperiencing, avoidance, hyperarousal, mental fatigue, sleep disturbance, somatic symptoms, nightmares, depression, anxiety) were assessed in the 8 weeks after trauma exposure (a critical period for the development of chronic symptoms) [7]. We hypothesized that it would be possible to derive and validate keystroke behaviors associated with each APNS outcome (ie, trait biomarkers stable over time), and that changes in such behaviors could accurately predict worsening of or recovery from APNSs over time (ie, state symptoms measured at the current assessment and subject to fluctuation) as well as trait symptoms (stable over time). To enhance rigor and replicability, journal article reporting standards were used in this study [41].

## Methods

### Study Design and Setting

Data for these analyses were obtained from the AURORA (Advancing Understanding of Recovery After Trauma) study, a prospective observational cohort study of a diverse sample of trauma survivors recruited from EDs across the United States in the early aftermath of trauma. Most EDs were part of academic medical centers and concentrated in the Northeast and Midwest of the United States. The full methodology of the AURORA study has been published elsewhere [2]. AURORA enrollment began in September 2017 and continued through June 30, 2020. Individuals were eligible to participate if they presented to 1 of 27 EDs within the national AURORA network. Participants were recruited from EDs and followed using a variety of remote follow-up procedures, including smartphone-based surveys that collected self-reported APNS data over 8 weeks after trauma exposure, and an app that collected keystroke data (SonderMind; formerly Mindstrong).

### Participants and Sample Size

Participants were recruited if they presented to an ED within the AURORA network after experiencing qualifying traumatic events (ie, MVC, physical assault, sexual assault, fall >10 ft, or mass casualty incidents). Participants were included if they presented within 72 hours of the trauma, were aged 18-75, and were able to speak and read English. Individuals were excluded if they had a solid organ injury grade >1 per the American Association for the Surgery of Trauma, significant hemorrhage, required operative intervention, or were likely to be admitted for >72 hours. A total of 3445 patients met these criteria, provided informed consent, and completed ED assessments. Of the 3445 patients enrolled at baseline, 2626 remained enrolled for at least 67 days or completed 90% or more of the week 8 survey and did not become pregnant or incarcerated. Of the 2626 participants, 1072 were included in this analysis because they had an Android operating system on their device, had keystroke data, and completed APNS measures. Only Android users were included because iOS users could opt out of the passive collection of keyboard behaviors, resulting in a lack of keystroke data (of note, Android users could also opt out of the app portion of the study while still participating in the overall study, if desired). See Tables S1 and S2 in Multimedia Appendix 1 for a comparison of Android versus iOS users. In brief, there were no clinically significant differences between Android and iOS users; however, Android users were, on average, 7 years older, less likely to be women, more racially and ethnically diverse, less educated, more likely to have been married, and more likely to have experienced traumas other than an MVC. Purposive sampling was used to recruit trauma survivors presenting at EDs, and the sample size for the parent study was determined based on power analysis for its aims [2].

Participants were, on average, 40 (SD 13) years of age (Table 1), with a slight majority being women (616/1072, 57.46%). Most participants self-identified as non–Hispanic Black (565/1072, 52.71%), followed by non–Hispanic White (353/1072, 32.93%), Hispanic (114/1072, 10.63%), and other (34/1072, 3.17%). Most reported having some college education (457/1072, 42.63%), a total annual family income of less than US $35,000 (653/1072, 60.91%), and being single/never married (578/1072, 53.92%).

**Table 1:** Sample demographic and trauma characteristics of trauma survivors participating in an observational cohort study (N=1072).

Characteristics		Values
Age (years), mean (SD)		39.4 (12.6)
Female, n (%)		616 (57.46)
**Total family income (US $), n (%)**		
	≤19K	348 (32.46)
	19,000-35,000	305 (28.45)
	35,001-50,000	129 (12.03)
	>50,000	174 (16.23)
**Race, n (%)**		
	Hispanic	114 (10.63)
	Non–Hispanic White	353 (32.93)
	Non–Hispanic Black	565 (53.71)
	Non–Hispanic other	34 (3.17)
**Education status, n (%)**		
	High school or less	422 (39.37)
	Some college	457 (42.63)
	College or more	189 (17.63)
**Marital status, n (%)**		
	Married	245 (22.85)
	Separated, divorced, widowed, or annulled	238 (22.20)
	Never been married	578 (53.92)
**Trauma type, n (%)**		
	Motor vehicle collision	772 (72.01)
	Physical assault	118 (11.01)
	Sexual assault	4 (0.37)
	Fall	83 (7.74)
	Nonmotorized collision	21 (1.96)
	Animal-related	20 (1.87)
	Other (including poisoning, burns, mass trauma exposure)	54 (5.04)

### Assessments and Data Sources

#### APNS Data Collection and Preparation

Sociodemographic characteristics measured in the ED were assessed via survey items [2]. Following the ED visit, participants completed a rotating battery of smartphone-based questionnaires consisting of brief assessments of 10 common APNS domains: pain [42], depression and anxiety symptoms [43], sleep [44], nightmares [45], somatic symptoms [46], difficulty with concentration/thinking/fatigue (mental fatigue) [46], and reexperiencing, avoidance, and hyperarousal [47]. Each survey item was administered at 6 time points within the first 8 weeks after trauma exposure using the app. Survey items, and the study day on which each item was administered, are presented in Table S3 in Multimedia Appendix 1.

These survey items were used as indicator variables to develop measurement models for each APNS domain, and factor scores for each symptom were computed for each participant at every time point. Joint measurement models that included all 6 time points within the first 8 weeks after trauma exposure were developed to define each symptom domain. Temporal correlations among these indicator variables were introduced to improve model fit when temporal autocorrelations were not fully explained by the joint measurement model. Model fit indices (eg, Comparative Fit Index, Tucker-Lewis Index, standardized root mean square residual) were used to evaluate the fit of each measurement model [48].

#### Keystroke Data Collection

Longitudinal keystroke behavior data were collected from an app installed on participants’ phones after trauma exposure (SonderMind; formerly Mindstrong). Keystroke data were collected from any smartphone use across apps and were not limited to study-specific interactions or the use of any specific app. Naturally occurring typing was captured, and users did not receive prompts to type. Typing word content was also collected and is reported in a separate manuscript [24]. Several aspects of keystroke behavior were collected: typing speed (the time elapsed between typing one character [or making one action] and the next); change of operations (switching from one process [eg, typing characters] to another [eg, deleting]); and scrolling (in any context, the distance scrolled in a list before clicking on an item). The data were processed within the app; thus, only the features reported in this manuscript were available for analysis.

### Ethical Considerations

The AURORA study protocol was approved by the Institutional Review Board (approval number 17-0703) at the University of North Carolina at Chapel Hill. All participants provided informed consent at the time of enrollment during their ED visit. All data reported are fully deidentified and anonymous. Participants were compensated for their involvement as follows: US $60 for the ED assessment, US $30 for app installation, and US $5 per serial assessment. No identification of individual participants or users in any images in the manuscript or multimedia appendices is possible. Shared data from the AURORA study are deidentified, with no patient identifiers included.

### Data Analysis: Keystroke Feature Extraction

The passive data collection app collected 41 features derived from participants’ interactions with their smartphones. Each feature had 23 signals, resulting in a total of 943 possible feature variables. Analyses began by pairing keystroke features with APNS data. Daily keystroke feature variables were merged with each of the 10 APNS constructs, whereby keystroke data collected on the same day or the day before the flash survey day were retained. The means of the keystroke feature variables across the 2 days were used to summarize the data in relation to the relevant APNS construct.

Second, within- and between-person correlations of keystroke features with each of the 10 APNS constructs were calculated. The top 50 variables with the highest absolute correlation values (either within- or between-persons) that were statistically significant (adjusted *P* value <.05, controlling for false discovery rate) were selected.

Third, the aggregated data were randomly divided into 2 equal parts: 1 for biomarker identification and 1 for validation. A bivariate linear mixed model approach was used to model the cross-sectional and longitudinal associations with each of the 10 APNS constructs. Feature variables that had significant associations (either cross-sectional or longitudinal) with any of the APNS constructs were then validated using the same bivariate linear mixed model in the remaining 50% of the data. Multiple testing was controlled by adjusting *P* values with a false discovery rate for the identification step and a Bonferroni correction for the validation step.

Fourth, we conducted an additional analysis for features that passed the identification and validation steps. For state biomarkers, we evaluated how accurately they predicted the direction of change in the corresponding APNS constructs (eg, worsening vs recovery).

Missing values for these 10 APNS constructs ranged from 250 out of 2438 (10.25%) to 285 out of 2554 (11.16%) during the first week after enrollment and from 1251 out of 2547 (49.12%) to 1479 out of 2438 (60.66%) around 6 months after enrollment. To explore the potential impact of missing data, we evaluated correlations between main outcomes (eg, posttraumatic stress disorder and pain) at different time points (eg, before trauma, 2 weeks, and 8 weeks after trauma) and completion rates of 4 main tasks (eg, watch wearing, flash survey, neurocognitive tests, and full surveys). All correlations were weak (<0.1; see Table S8 in Multimedia Appendix 1), suggesting that missing data were not driven by these outcomes. Thus, missing data for these APNS construct scores were considered missing at random and were imputed using the joint measurement model.

Of note, due to the large number of highly intracorrelated features associated with various APNSs, results were simplified as follows. For similar signal processing techniques (eg, measures of central tendency such as mean and median; first-, second-, and third-order spectral moments) that were highly intracorrelated with one another (*r*s>0.80), only the signal processing technique variable with the highest correlation with the relevant APNS was retained in the results and tables (eg, if both mean and median frequency were associated with pain, only mean frequency was retained as a predictor when it had the higher correlation with pain). Full results, including all variables, are available in Tables S4-S7 in Multimedia Appendix 1.

## Results

### Participant Flow and Sociodemographic, Trauma Exposure, and Clinical Characteristics

The final sample consisted of 1072 trauma survivors who used the passive data collection app on their Android smartphones (see Figure S1 in Multimedia Appendix 1). The majority of participants experienced an MVC as their trauma type (772/1072, 72.01%), followed by physical assault (118/1072, 11.01%), fall >10 ft (83/1072, 7.74%), other (54/1072, 5.04%; eg, poisoning, burn, mass trauma), nonmotorized collision (21/1072, 1.96%), animal-related (20/1072, 1.87%), and sexual assault (4/1072, 0.37%).

### Derivation of Trait Biomarkers

First, we evaluated whether trait-level keystroke biomarkers could identify individuals experiencing high levels of specific APNS (Table 2) at the trait level (ie, symptoms stable over time). Within- and between-person correlations indicated that a total of 24 unique keystroke biomarkers related to typing speed were associated with pain (Table 2; absolute values of *r*s=0.22-0.25, *P*s=.02; α level set at *P*<.05, applied to Bonferroni-adjusted *P* values for these and all analyses), indicating that, overall, slower character typing speed was associated with higher levels of pain. Additionally, 1 keystroke biomarker was associated with increased reexperiencing symptoms (ie, slower speed when typing the space bar; *r*=0.18, *P*=.047). Finally, 5 scroll-related biomarkers were associated with mental fatigue (*r*s=0.18-0.19, *P*s=.031-.047), indicating that slower scrolling was associated with higher levels of mental fatigue. Correlations between biomarkers and specific APNS were in the small- to medium-effect size range and were statistically significant at *P*<.05 after Bonferroni adjustment.

**Table 2:** Trait keystroke biomarkers captured via the app and associations with adverse posttraumatic neuropsychiatric sequelae among trauma survivors participating in an observational cohort study.

Construct and theme			Type		Signal processing		Correlation (*r*)		Bonferroni-adjusted *P* value
**Pain**									
	Typing speed^a^	Two characters in a row		SD		0.22		.02	
	Typing speed	Two characters in a row		Mean log		0.24		.02	
	Typing speed	Two characters in a row		80th percentile		0.24		.02	
	Typing speed	Two characters in a row		Total (sum) power		–0.25		.02	
	Typing speed	Two characters in a row		First spectral moment		–0.25		.02	
	Typing speed	Two characters		Mean log		0.25		.02	
	Typing speed	Two characters		10th percentile		0.24		.02	
	Typing speed	Two characters		Total (sum) power		–0.25		.02	
	Typing speed	Two characters		Mean frequency		–0.25		.02	
	Typing speed	Two characters		First spectral moment		–0.25		.02	
	Typing speed	Two characters		Second spectral moment		–0.25		.02	
	Typing speed	Two characters		Mean log		0.24		.02	
	Typing speed	Two characters		50th percentile		0.25		.02	
	Typing speed	Two characters		Total (sum) power		–0.25		.02	
	Typing speed	Two characters		First spectral moment		–0.25		.02	
	Typing speed	Three characters or fewer in a row		Mean log		0.24		.02	
	Typing speed	Three characters or fewer in a row		50th percentile		0.24		.02	
	Typing speed	Three characters or fewer in a row		Total (sum) power		–0.25		.02	
	Typing speed	Three characters or fewer in a row		Second spectral moment		–0.25		.02	
	Typing speed	More than 3 characters in a row		Mean		0.24		.02	
	Typing speed	More than 3 characters in a row		Mean log		0.25		.02	
	Typing speed	More than 3 characters in a row		80th percentile		0.25		.02	
	Typing speed	More than 3 characters in a row		Total (sum) power		–0.26		.02	
	Typing speed	More than 3 characters in a row		First spectral moment		–0.26		.02	
**Reexperiencing**									
	Change of operations^b^	Character to space		Mean power		–0.18		.047	
**Mental fatigue**									
	Scroll^c^	Scroll then click		Mean		0.18		.047	
	Scroll	Scroll then click		Mean log		0.19		.03	
	Scroll	Scroll then click		*a* (intercept of linear regression of sorted time differences)		0.18		.047	
	Scroll	Scroll then click		Total (sum) power		–0.19		.03	
	Scroll	Scroll then click		Third spectral moment		–0.19		.03	

^a^Typing speed is the length of time elapsed from typing 1 character (or making 1 action) to another (higher values=slower typing speed).

^b^Change of operations refers to switching from 1 process (eg, typing characters) to another (eg, deleting; thought to measure task or set shifting).

^c^Scroll refers to the distance scrolled in a list before clicking on the item they are looking for, or the speed in scrolling through a list (thought to measure processing speed). When the keystroke type description includes “characters in a row,” this refers to typing several characters in sequence that are not interrupted by a space key press, deletion, or other action. Correlation can be used as a measure of effect size for linear relationships, where a positive/negative correlation of *r* between variables *X* and *Y* indicates that a 1 SD change in *X* is associated with *r* SD change in *Y* in the same/opposite direction.

### Derivation of State Biomarkers

Second, we evaluated whether changes in keystroke behaviors were associated with worsening or recovery of state-level (ie, measured at the current assessment and subject to fluctuations) specific APNS during the initial 8 weeks after trauma using bivariate linear mixed models (Table 3). State-level biomarkers were identified for changes in pain, sleep disturbance, hyperarousal, reexperiencing, somatic symptoms, and mental fatigue. For pain, 18 separate keystroke-related biomarkers were identified (*r*s=0.07-0.10, *P*s=.02; 17 related to typing speed and 1 related to scrolling speed). Overall, slower typing and scrolling speeds were associated with higher levels of pain. For sleep disturbance, 11 state biomarkers related to typing speed were identified, with absolute *r* values ranging from 0.07 to 0.09 (*P*s=.02). Again, slower typing speed was associated with increased sleep disturbance. For somatic symptoms, 19 typing speed–related biomarkers were identified (*r*s=0.02), along with 4 biomarkers associated with mental fatigue (2 related to scrolling speed and 2 related to typing speed; *r*s=0.02-0.04). Overall, slower scrolling and typing speeds were associated with increased somatic symptoms and mental fatigue.

**Table 3:** State keystroke biomarkers captured via the app and associations with adverse posttraumatic neuropsychiatric sequelae among trauma survivors participating in an observational cohort study.

Construct and theme			Type		Signal processing		Correlation (*r*)		Bonferroni-adjusted *P* value
**Pain**									
	Typing speed^a^	Two characters in a row		Total (sum) power		–0.07		.02	
	Typing speed	Two characters in a row		Third spectral moment		–0.09		.02	
	Typing speed	Two characters		Total (sum) power		–0.07		.02	
	Typing speed	Two characters		Mean frequency		–0.09		.02	
	Typing speed	Two characters		Third spectral moment		–0.08		.02	
	Typing speed	Two characters		Total (sum) frequency		–0.07		.02	
	Typing speed	Two characters		Mean frequency		–0.09		.02	
	Typing speed	Two characters		Third spectral moment		–0.09		.02	
	Typing speed	Consecutive typing events		Mean log		0.08		.02	
	Typing speed	Consecutive typing events		50th percentile		0.07		.02	
	Typing speed	Consecutive typing events		Total (sum) power		–0.09		.02	
	Typing speed	Consecutive typing events		Median frequency		–0.08		.02	
	Typing speed	Consecutive typing events		Mean frequency		–0.10		.02	
	Typing speed	Consecutive typing events		Third spectral moment		–0.10		.02	
	Scroll^b^	Scroll twice to item		Maximum fractal length		–0.09		.02	
	Typing speed	Three characters or fewer in a row		Mean frequency		–0.07		.02	
	Typing speed	More than 3 characters in a row		Mean frequency		–0.07		.02	
	Typing speed	More than 3 characters in a row		Third spectral moment		–0.07		.02	
**Sleep disturbance**									
	Typing speed	Two characters in a row		First spectral moment		–0.07		.02	
	Typing speed	Two characters		Mean log		0.07		.02	
	Typing speed	Two characters		Second spectral moment		–0.07		.02	
	Typing speed	Two characters		First spectral moment		–0.07		.02	
	Typing speed	Consecutive typing events		Mean log		0.08		.02	
	Typing speed	Consecutive typing events		Total (sum) power		–0.08		.02	
	Typing speed	Consecutive typing events		Mean frequency		–0.08		.02	
	Typing speed	Consecutive typing events		Second spectral moment		–0.08		.02	
	Typing speed	More than 3 characters in a row		Mean		0.08		.02	
	Typing speed	More than 3 characters in a row		80th percentile		0.08		.02	
	Typing speed	More than 3 characters in a row		Mean power		–0.09		.02	
**Hyperarousal**									
	Typing speed	Character to space		Second spectral moment		–0.07		.02	
	Typing speed	Two characters in a row		Mean frequency		–0.09		.02	
	Typing speed	Two characters in a row		First spectral moment		–0.10		.02	
	Typing speed	Two characters		First spectral moment		–0.09		.02	
	Typing speed	Consecutive typing events		Second spectral moment		–0.09		.02	
	Typing speed	More than 3 characters in a row		20th percentile		0.07		.02	
	Typing speed	More than 3 characters in a row		Total (sum) power		–0.08		.02	
	Typing speed	More than 3 characters in a row		First spectral moment		–0.08		.02	
	Typing speed	The second set of at least 5 characters in a row		10th percentile		0.07		.02	
	Typing speed	The second set of at least 5 characters in a row		80th percentile		0.08		.02	
	Typing speed	The second set of at least 5 characters in a row		Total (sum) power		–0.08		.02	
	Typing speed	The second set of at least 5 characters in a row		Mean frequency		–0.07		.02	
	Typing speed	The second set of at least 5 characters in a row		First spectral moment		–0.08		.02	
**Reexperiencing**									
	Typing speed	Character to space		90th percentile		0.07		.04	
	Typing speed	Character to space		Second spectral moment		–0.08		.02	
	Typing speed	Character to space		Median frequency		–0.07		.02	
	Typing speed	Consecutive typing events		Mean log		0.07		.02	
	Typing speed	Consecutive typing events		Third spectral moment		–0.09		.02	
	Typing speed	Three characters or fewer in a row		Total (sum) power		–0.09		.02	
	Typing speed	Four characters or fewer, then space		Mean frequency		–0.07		.02	
**Somatic symptoms**									
	Typing speed	Two characters in a row		Total (sum) power		–0.08		.02	
	Typing speed	Two characters in a row		Mean frequency		–0.08		.02	
	Typing speed	Two characters in a row		Second spectral moment		–0.08		.02	
	Typing speed	Two characters		Total (sum) power		–0.07		.02	
	Typing speed	Two characters		Mean frequency		–0.08		.02	
	Typing speed	Two characters		Third spectral moment		–0.08		.02	
	Typing speed	Two characters		Median frequency		–0.08		.02	
	Typing speed	Two characters		Third spectral moment		–0.09		.02	
	Typing speed	Consecutive typing events		80th percentile		0.07		.02	
	Typing speed	Consecutive typing events		Total (sum) power		–0.08		.02	
	Typing speed	Consecutive typing events		Mean frequency		–0.09		.02	
	Typing speed	Consecutive typing events		Second spectral moment		–0.08		.02	
	Typing speed	Three characters or fewer in a row		Total (sum) power		–0.06		.04	
	Typing speed	Three characters or fewer in a row		Second spectral moment		–0.07		.02	
	Typing speed	More than 3 characters in a row		Mean log		0.07		.02	
	Typing speed	More than 3 characters in a row		50th percentile		0.07		.02	
	Typing speed	More than 3 characters in a row		Total (sum) power		–0.09		.02	
	Typing speed	More than 3 characters in a row		Mean frequency		–0.10		.02	
	Typing speed	More than 3 characters in a row		Second spectral moment		–0.10		.02	
**Mental fatigue**									
	Scroll	Speed of scroll to item		50th percentile		0.06		.04	
	Scroll	Speed of scroll to item		Median frequency		–0.07		.02	
	Typing speed	More than 3 characters in a row		Mean frequency		–0.07		.02	
	Typing speed	More than 3 characters in a row		Third spectral moment		–0.07		.02	

^a^Typing speed is the length of time elapsed from typing 1 character (or making 1 action) to another (higher values=slower typing speed).

^b^Scroll refers to the distance scrolled in a list before clicking on the item they are looking for, or the speed in scrolling through a list (thought to measure processing speed). When the keystroke type description includes “characters in a row,” this refers to typing several characters in sequence that are not interrupted by a space key press, deletion, or other action. Correlation can be used as a measure of effect size for linear relationships, where a positive/negative correlation of *r* between variables *X* and *Y* indicates that a 1 SD change in *X* is associated with *r* SD change in *Y* in the same/opposite direction. Regarding PTS, 13 typing speed–related biomarkers were associated with state changes in hyperarousal symptoms (*r*s=.02-.04), and 7 typing speed–related biomarkers were associated with state changes in reexperiencing symptoms (*r*s=.02-.04). In general, slower typing speed was associated with elevated PTS. All correlations for state biomarkers were in the small effect size range and were statistically significant at *P*<.05 after Bonferroni adjustment.

### Utility of State Biomarkers

Finally, we examined the potential utility of keystroke biomarkers as screening tools for worsening or improving APNSs after trauma using bivariate linear mixed models (Tables 4 and 5; only statistically significant biomarkers are presented). Worsening and improvement in self-report symptoms were defined as symptom severity at 6 months minus symptom severity in the first week >0 and <0, respectively. High positive predictive values for symptom recovery and negative predictive values for worsening suggest that simple keystroke measures, passively collected via smartphone, may have utility as initial screening tools for mental fatigue, pain, and somatic symptom outcomes among diverse trauma survivors.

Specifically, for identifying worsening PTS symptoms, we found that 11 typing speed–related biomarkers were associated with hyperarousal symptoms, and 7 typing speed–related biomarkers were associated with reexperiencing symptoms. A total of 2 scroll-related and 2 typing speed–related biomarkers were associated with worsening mental fatigue. For pain, 16 typing speed–related and 1 scroll-related biomarker were associated with worsening symptoms. Eleven typing speed–related biomarkers were predictive of worsening sleep. Finally, 19 typing speed–related biomarkers were predictive of worsening somatic symptoms.

**Table 4:** Prediction of worsening adverse posttraumatic neuropsychiatric symptoms using state keystroke biomarkers captured via the app among trauma survivors participating in an observational cohort study.

Construct and worsening, *n* (%)			Theme		Type		Signal processing		Sensitivity		Specificity		Positive predictive value		Negative predictive value
**Hyperarousal (N=509)**															
	263 (51.7)	Typing speed^a^		Character to space		Second spectral moment		0.52		0.48		0.53		0.47	
	263 (51.7)	Typing speed		Two characters in a row		First spectral moment		0.51		0.49		0.52		0.49	
	263 (51.7)	Typing speed		Two characters		First spectral moment		0.5		0.5		0.52		0.48	
	263 (51.7)	Typing speed		Consecutive typing events		Second spectral moment		0.5		0.5		0.52		0.48	
	263 (51.7)	Typing speed		More than 3 characters in a row		20th percentile		0.51		0.47		0.51		0.47	
	263 (51.7)	Typing speed		More than 3 characters in a row		Total (sum) power		0.54		0.47		0.53		0.48	
	263 (51.7)	Typing speed		More than 3 characters in a row		First spectral moment		0.54		0.47		0.53		0.48	
	263 (51.7)	Typing speed		The second set of at least 5 characters in a row		80th percentile		0.53		0.52		0.59		0.46	
	263 (51.7)	Typing speed		The second set of at least 5 characters in a row		Total (sum) power		0.51		0.48		0.57		0.43	
	263 (51.7)	Typing speed		The second set of at least 5 characters in a row		Mean frequency		0.53		0.49		0.58		0.44	
	263 (51.7)	Typing speed		The second set of at least 5 characters in a row		First spectral moment		0.51		0.48		0.56		0.43	
**Mental fatigue (N=574)**															
	160 (27.9)	Scroll^b^		Speed of scroll to item		50th percentile		0.54		0.5		0.3		0.74	
	160 (27.9)	Scroll		Speed of scroll to item		Median frequency		0.56		0.5		0.3		0.75	
	160 (27.9)	Typing speed		More than 3 characters in a row		Mean frequency		0.49		0.56		0.3		0.74	
	160 (27.9)	Typing speed		More than 3 characters in a row		Third spectral moment		0.54		0.55		0.32		0.76	
**Pain (N=692)**															
	159 (23.0)	Typing speed		Two characters in a row		Total (sum) power		0.41		0.61		0.24		0.78	
	159 (23.0)	Typing speed		Two characters in a row		Third spectral moment		0.4		0.6		0.23		0.77	
	159 (23.0)	Typing speed		Two characters		Total (sum) power		0.39		0.59		0.22		0.77	
	159 (23.0)	Typing speed		Two characters		Mean frequency		0.36		0.65		0.23		0.77	
	159 (23.0)	Typing speed		Two characters		Third spectral moment		0.38		0.62		0.23		0.77	
	159 (23.0)	Typing speed		Two characters		Total (sum) power		0.43		0.62		0.25		0.79	
	159 (23.0)	Typing speed		Two characters		Mean frequency		0.36		0.62		0.22		0.76	
	159 (23.0)	Typing speed		Two characters		Third spectral moment		0.41		0.63		0.25		0.78	
	159 (23.0)	Typing speed		Consecutive typing events		Mean log		0.37		0.65		0.24		0.78	
	159 (23.0)	Typing speed		Consecutive typing events		50th percentile		0.36		0.64		0.23		0.77	
	159 (23.0)	Typing speed		Consecutive typing events		Total (sum) power		0.39		0.61		0.23		0.77	
	159 (23.0)	Typing speed		Consecutive typing events		Mean frequency		0.39		0.62		0.23		0.77	
	159 (23.0)	Typing speed		Consecutive typing events		Third spectral moment		0.44		0.61		0.25		0.79	
	159 (23.0)	Scroll		Scroll twice to item		Maximum fractal length		0.36		0.59		0.21		0.75	
	159 (23.0)	Typing speed		Three characters or fewer in a row		Mean frequency		0.44		0.58		0.24		0.77	
	159 (23.0)	Typing speed		More than 3 characters in a row		Mean frequency		0.42		0.55		0.22		0.77	
	159 (23.0)	Typing speed		More than 3 characters in a row		Third spectral moment		0.46		0.54		0.23		0.77	
**Reexperiencing (N=479)**															
	226 (47.2)	Typing speed		Character to space		90th percentile		0.53		0.48		0.46		0.55	
	226 (47.2)	Typing speed		Character to space		Second spectral moment		0.55		0.49		0.48		0.56	
	226 (47.2)	Typing speed		Character to space		Median frequency		0.55		0.54		0.5		0.59	
	226 (47.2)	Typing speed		Consecutive typing events		Mean log		0.51		0.46		0.46		0.52	
	226 (47.2)	Typing speed		Consecutive typing events		Third spectral moment		0.5		0.5		0.47		0.53	
	226 (47.2)	Typing speed		Three characters or fewer in a row		Total (sum) power		0.54		0.51		0.49		0.55	
	226 (47.2)	Typing speed		Four characters or fewer, then space		Mean frequency		0.56		0.47		0.48		0.56	
**Sleep (N=487)**															
	269 (55.2)	Typing speed		Two characters in a row		First spectral moment		0.51		0.49		0.55		0.45	
	269 (55.2)	Typing speed		Two characters		Mean log		0.51		0.5		0.55		0.45	
	269 (55.2)	Typing speed		Two characters		Second spectral frequency		0.5		0.48		0.54		0.44	
	269 (55.2)	Typing speed		Two characters		First spectral frequency		0.53		0.5		0.57		0.47	
	269 (55.2)	Typing speed		Consecutive typing events		Mean log		0.52		0.52		0.57		0.47	
	269 (55.2)	Typing speed		Consecutive typing events		Total (sum) power		0.51		0.51		0.56		0.46	
	269 (55.2)	Typing speed		Consecutive typing events		Mean frequency		0.52		0.51		0.57		0.46	
	269 (55.2)	Typing speed		Consecutive typing events		Second spectral moment		0.51		0.51		0.56		0.46	
	269 (55.2)	Typing speed		More than 3 characters in a row		Mean		0.52		0.48		0.56		0.44	
	269 (55.2)	Typing speed		More than 3 characters in a row		80th percentile		0.5		0.5		0.56		0.44	
	269 (55.2)	Typing speed		More than 3 characters in a row		Mean power		0.5		0.46		0.54		0.42	
**Somatic symptoms (N=572)**															
	130 (22.7)	Typing speed		Two characters in a row		Total (sum) power		0.46		0.54		0.23		0.77	
	130 (22.7)	Typing speed		Two characters in a row		Mean frequency		0.48		0.57		0.25		0.79	
	130 (22.7)	Typing speed		Two characters in a row		Second spectral moment		0.45		0.53		0.22		0.77	
	130 (22.7)	Typing speed		Two characters		Total (sum) power		0.48		0.52		0.23		0.78	
	130 (22.7)	Typing speed		Two characters		Mean frequency		0.42		0.54		0.21		0.76	
	130 (22.7)	Typing speed		Two characters		Third spectral moment		0.48		0.53		0.23		0.78	
	130 (22.7)	Typing speed		Two characters		Median frequency		0.45		0.54		0.22		0.77	
	130 (22.7)	Typing speed		Two characters		Third spectral moment		0.49		0.54		0.24		0.78	
	130 (22.7)	Typing speed		Consecutive typing events		80th percentile		0.47		0.54		0.23		0.78	
	130 (22.7)	Typing speed		Consecutive typing events		Total (sum) power		0.48		0.56		0.25		0.79	
	130 (22.7)	Typing speed		Consecutive typing events		Mean frequency		0.43		0.54		0.22		0.76	
	130 (22.7)	Typing speed		Consecutive typing events		Second spectral moment		0.47		0.57		0.24		0.79	
	130 (22.7)	Typing speed		Three characters or fewer in a row		Total (sum) power		0.49		0.56		0.25		0.79	
	130 (22.7)	Typing speed		Three characters or fewer in a row		Second spectral moment		0.5		0.55		0.25		0.79	
	130 (22.7)	Typing speed		More than 3 characters in a row		Mean log		0.54		0.55		0.26		0.8	
	130 (22.7)	Typing speed		More than 3 characters in a row		50th percentile		0.54		0.53		0.25		0.8	
	130 (22.7)	Typing speed		More than 3 characters in a row		Total (sum) power		0.53		0.54		0.26		0.8	
	130 (22.7)	Typing speed		More than 3 characters in a row		Mean frequency		0.52		0.57		0.26		0.8	
	130 (22.7)	Typing speed		More than 3 characters in a row		Second spectral moment		0.54		0.55		0.26		0.8	

^a^Typing speed is the length of time elapsed from typing 1 character (or making 1 action) to another (higher values=slower typing speed).

^b^Scroll refers to the distance scrolled in a list before clicking on the item they are looking for, or the speed in scrolling through a list (thought to measure processing speed). When the keystroke type description includes “characters in a row,” this refers to typing several characters in sequence that are not interrupted by a space key press, deletion, or other action.

**Table 5:** Prediction of recovering adverse posttraumatic neuropsychiatric symptoms using state keystroke biomarkers captured via the app among trauma survivors participating in an observational cohort study.

Construct and recovering, n (%)		Theme	Type	Signal processing	Sensitivity	Specificity	Positive predictive value	Negative predictive value
**Hyperarousal (N=509)**								
	246 (48.3)	Typing speed^a^	Character to space	Second spectral moment	0.52	0.48	0.47	0.53
	246 (48.3)	Typing speed	Two characters in a row	First spectral moment	0.51	0.49	0.48	0.51
	246 (48.3)	Typing speed	Two characters	First spectral moment	0.5	0.5	0.48	0.52
	246 (48.3)	Typing speed	Consecutive typing events	Second spectral moment	0.5	0.5	0.48	0.52
	246 (48.3)	Typing speed	More than 3 characters in a row	20th percentile	0.53	0.49	0.49	0.53
	246 (48.3)	Typing speed	More than 3 characters in a row	Total (sum) power	0.53	0.46	0.47	0.52
	246 (48.3)	Typing speed	More than 3 characters in a row	First spectral moment	0.53	0.46	0.47	0.52
	246 (48.3)	Typing speed	The second set of at least 5 characters in a row	80th percentile	0.48	0.47	0.41	0.54
	246 (48.3)	Typing speed	The second set of at least 5 characters in a row	Total (sum) power	0.52	0.49	0.43	0.57
	246 (48.3)	Typing speed	The second set of at least 5 characters in a row	Mean frequency	0.51	0.47	0.42	0.56
	246 (48.3)	Typing speed	The second set of at least 5 characters in a row	First spectral moment	0.52	0.49	0.44	0.57
**Mental fatigue (N=573)**								
	413 (72.1)	Scroll^b^	Speed of scroll to item	50th percentile	0.5	0.46	0.7	0.26
	413 (72.1)	Scroll	Speed of scroll to item	Median frequency	0.5	0.44	0.7	0.25
	413 (72.1)	Typing speed	More than 3 characters in a row	Mean frequency	0.44	0.51	0.7	0.26
	413 (72.1)	Typing speed	More than 3 characters in a row	Third spectral moment	0.45	0.46	0.68	0.24
**Pain (N=692)**								
	533 (77.0)	Typing speed	Two characters in a row	Total (sum) power	0.39	0.59	0.76	0.22
	533 (77.0)	Typing speed	Two characters in a row	Third spectral moment	0.4	0.6	0.77	0.23
	533 (77.0)	Typing speed	Two characters	Total (sum) power	0.41	0.61	0.78	0.23
	533 (77.0)	Typing speed	Two characters	Mean frequency	0.35	0.64	0.77	0.23
	533 (77.0)	Typing speed	Two characters	Third spectral moment	0.38	0.62	0.77	0.23
	533 (77.0)	Typing speed	Two characters	Total (sum) power	0.38	0.57	0.75	0.21
	533 (77.0)	Typing speed	Two characters	Mean frequency	0.38	0.64	0.78	0.24
	533 (77.0)	Typing speed	Two characters	Third spectral moment	0.37	0.59	0.75	0.22
	533 (77.0)	Typing speed	Consecutive typing events	Mean log	0.35	0.63	0.76	0.23
	533 (77.0)	Typing speed	Consecutive typing events	50th percentile	0.36	0.64	0.77	0.23
	533 (77.0)	Typing speed	Consecutive typing events	Total (sum) power	0.39	0.61	0.77	0.23
	533 (77.0)	Typing speed	Consecutive typing events	Mean frequency	0.38	0.61	0.77	0.23
	533 (77.0)	Typing speed	Consecutive typing events	Third spectral moment	0.39	0.56	0.75	0.21
	533 (77.0)	Scroll	Scroll twice to item	Maximum fractal length	0.41	0.64	0.79	0.25
	533 (77.0)	Typing speed	Three characters or fewer in a row	Mean frequency	0.42	0.56	0.76	0.23
	533 (77.0)	Typing speed	More than 3 characters in a row	Mean frequency	0.45	0.58	0.78	0.23
	533 (77.0)	Typing speed	More than 3 characters in a row	Third spectral moment	0.46	0.54	0.77	0.23
**Reexperiencing (N=479)**								
	253 (52.8)	Typing speed	Character to space	90th percentile	0.52	0.47	0.54	0.45
	253 (52.8)	Typing speed	Character to space	Second spectral moment	0.51	0.45	0.52	0.44
	253 (52.8)	Typing speed	Character to space	Median frequency	0.46	0.45	0.5	0.41
	253 (52.8)	Typing speed	Consecutive typing events	Mean log	0.54	0.49	0.54	0.48
	253 (52.8)	Typing speed	Consecutive typing events	Third spectral moment	0.5	0.5	0.53	0.47
	253 (52.8)	Typing speed	Three characters or fewer in a row	Total (sum) power	0.49	0.46	0.51	0.45
	253 (52.8)	Typing speed	Four characters or fewer, then space	Mean frequency	0.53	0.44	0.52	0.44
**Sleep (N=487)**								
	218 (44.8)	Typing speed	Two characters in a row	First spectral moment	0.51	0.49	0.45	0.55
	218 (44.8)	Typing speed	Two characters	Mean log	0.5	0.49	0.45	0.55
	218 (44.8)	Typing speed	Two characters	Second spectral moment	0.52	0.5	0.46	0.56
	218 (44.8)	Typing speed	Two characters	First spectral moment	0.5	0.47	0.43	0.53
	218 (44.8)	Typing speed	Consecutive typing events	Mean log	0.48	0.48	0.43	0.53
	218 (44.8)	Typing speed	Consecutive typing events	Total (sum) power	0.49	0.49	0.44	0.54
	218 (44.8)	Typing speed	Consecutive typing events	Mean frequency	0.49	0.48	0.43	0.54
	218 (44.8)	Typing speed	Consecutive typing events	Second spectral moment	0.49	0.49	0.44	0.54
	218 (44.8)	Typing speed	More than 3 characters in a row	Mean	0.52	0.48	0.44	0.56
	218 (44.8)	Typing speed	More than 3 characters in a row	80th percentile	0.5	0.5	0.44	0.56
	218 (44.8)	Typing speed	More than 3 characters in a row	Mean power	0.54	0.5	0.46	0.58
**Somatic symptoms (N=572)**								
	442 (77.3)	Typing speed	Two characters in a row	Total (sum) power	0.46	0.54	0.77	0.23
	442 (77.3)	Typing speed	Two characters in a row	Mean frequency	0.43	0.52	0.75	0.21
	442 (77.3)	Typing speed	Two characters in a row	Second spectral moment	0.47	0.55	0.78	0.23
	442 (77.3)	Typing speed	Two characters	Total (sum) power	0.48	0.52	0.77	0.22
	442 (77.3)	Typing speed	Two characters	Mean frequency	0.46	0.58	0.79	0.24
	442 (77.3)	Typing speed	Two characters	Third spectral moment	0.47	0.52	0.77	0.22
	442 (77.3)	Typing speed	Two characters	Median frequency	0.46	0.55	0.78	0.23
	442 (77.3)	Typing speed	Two characters	Third spectral moment	0.46	0.51	0.76	0.22
	442 (77.3)	Typing speed	Consecutive typing events	80th percentile	0.46	0.53	0.77	0.22
	442 (77.3)	Typing speed	Consecutive typing events	Total (sum) power	0.44	0.52	0.75	0.21
	442 (77.3)	Typing speed	Consecutive typing events	Mean frequency	0.46	0.57	0.78	0.24
	442 (77.3)	Typing speed	Consecutive typing events	Second spectral moment	0.43	0.53	0.76	0.21
	442 (77.3)	Typing speed	Three characters or fewer in a row	Total (sum) power	0.44	0.51	0.75	0.21
	442 (77.3)	Typing speed	Three characters or fewer in a row	Second spectral moment	0.45	0.5	0.75	0.21
	442 (77.3)	Typing speed	Fewer than 3 characters in a row	Mean log	0.45	0.46	0.74	0.2
	442 (77.3)	Typing speed	Fewer than 3 characters in a row	50th percentile	0.47	0.46	0.75	0.2
	442 (77.3)	Typing speed	Fewer than 3 characters in a row	Total (sum) power	0.46	0.47	0.74	0.2
	442 (77.3)	Typing speed	Fewer than 3 characters in a row	Mean frequency	0.43	0.48	0.74	0.2
	442 (77.3)	Typing speed	Fewer than 3 characters in a row	Second spectral moment	0.45	0.46	0.74	0.2

^a^Typing speed is the length of time elapsed from typing 1 character (or making 1 action) to another (higher values=slower typing speed).

^b^Scroll refers to the distance scrolled in a list before clicking on the item they are looking for, or the speed in scrolling through a list (thought to measure processing speed). When the keystroke type description includes “characters in a row,” this refers to typing several characters in sequence that are not interrupted by a space key press, deletion, or other action.

For identifying recovering PTS symptoms, 11 typing speed–related biomarkers were predictive of hyperarousal, and 7 typing speed–related biomarkers were associated with reexperiencing symptoms. Similarly, 2 scroll-related and 2 typing speed–related biomarkers were associated with recovery from mental fatigue. A total of 17 biomarkers (16 typing speed–related and 1 scroll-related) were predictive of recovery from pain. For recovery from sleep disturbance, there were 11 typing speed–related biomarkers. Finally, there were 19 typing speed–related biomarkers associated with recovery from somatic symptoms.

## Discussion

### Principal Findings

Millions of Americans present to emergency care each year following exposure to a traumatic stressor [49]. Although the majority recover from these events, a significant minority go on to develop chronic APNSs, including PTS, depression, anxiety, sleep disturbance, nightmares, pain, somatic symptoms, and mental fatigue [2]. The ability to identify these individuals and, ultimately, intervene to prevent the development of chronic APNSs would advance public health and reduce substantial personal suffering. Approximately 90% of Americans own a smartphone [30], and data from smartphone use can be collected passively in daily life with minimal effort from the user. This study is, to our knowledge, the first to suggest that such smartphone data may serve as biomarkers for the development of chronic APNSs in the 8 weeks after traumatic event exposure and presentation to emergency care. Further, keystroke data, which index cognitive and motor behavior, may be useful for tracking worsening and recovery of APNSs. Specifically, smartphone data measuring typing and scrolling behaviors during daily life among 1072 trauma survivors who presented for emergency care helped identify those with high levels of pain, reexperiencing, and mental fatigue in the 8 weeks after trauma exposure. In addition, smartphone indicators predicted state changes in pain, somatic symptoms, mental fatigue, sleep disturbance, and PTS (ie, reexperiencing and hyperarousal), including identifying individuals with worsening versus recovering symptoms. Effects were typically in the small- to medium-effect size range. Importantly, the sample was diverse, with more than half identifying as Black and socioeconomically disadvantaged, with a third reporting a total annual family income of less than US $19,000. This contrasts with other studies in the literature using smartphone biomarkers, which tend to include predominantly White, highly educated samples with higher socioeconomic status [33,50].

### Comparison With Prior Work

Regarding trait biomarkers, our results suggest that 18 keystroke biomarkers could identify individuals with elevated pain in the 8 weeks after trauma exposure. Specifically, individuals with slower typing speeds demonstrated higher levels of pain. Frequency-domain features (eg, total [sum] power, mean frequency, and spectral moments) were the most common signal processing techniques observed in typing speed–related biomarkers, suggesting that these techniques may be particularly useful. Change-of-operation behaviors (eg, time spent switching from typing to pressing the space bar) were associated with reexperiencing symptoms. This is consistent with prior research indicating that PTS symptoms may be associated with difficulties in set shifting [51,52]; however, this is the first study, to our knowledge, to extend such findings to passively collected smartphone data. Finally, scrolling behaviors were associated with mental fatigue, such that longer time spent scrolling to identify desired items was associated with higher levels of mental fatigue. Effects were all in the small- to medium-effect size range. These results are the first to suggest that keystroke biomarkers can identify trauma survivors with pain, PTS symptoms, and mental fatigue. However, the findings are consistent with prior studies in other populations [33], suggesting that keystroke behaviors, including slower typing speeds, can identify individuals with depressive symptoms [37], cognitive impairment [35,36], and pain and clinical disability [50]. These results also align with research indicating that other passively sensed data (eg, sleep and physiological metrics) may serve as potential biomarkers of posttraumatic stress disorder symptoms [39].

State keystroke biomarkers were also identified that could detect within-person changes in APNSs, suggesting improvement or worsening of symptoms. Changes in physical APNSs, such as pain, somatic symptoms, mental fatigue, and sleep disturbance, were predicted by typing and scrolling speed. Changes in mental health–related APNSs (ie, reexperiencing, hyperarousal) were predicted by typing speed. These findings are consistent with prior research indicating that changes in keystroke behaviors can predict within-person changes in mood [31-34]. However, the study findings are, to our knowledge, the first to suggest that keystroke biomarkers can predict worsening or recovery of APNSs among a large sample of trauma survivors followed over the acute posttrauma period.

**Figure 1:**
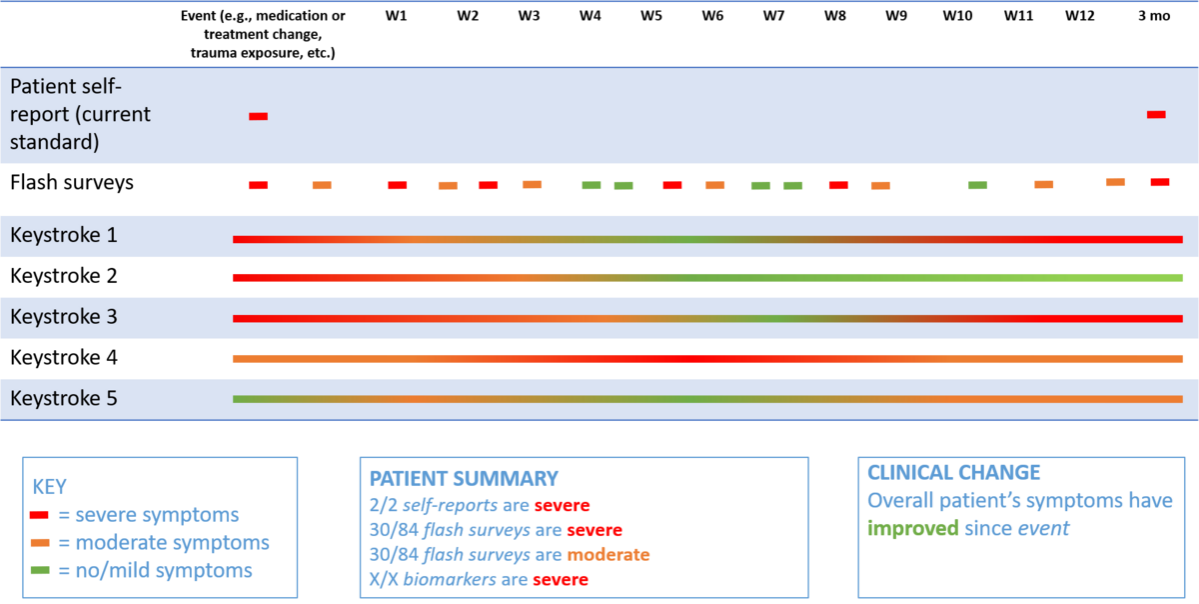
An example of a clinical tool that could be developed to leverage state data from passively collected biomarkers to complement assessments conducted by providers during clinical visits. mo: month; w: week.

This study has important clinical implications. Although the sensitivity and specificity of the results indicate that keystroke biomarkers may not be sufficient on their own, they could be useful as part of a suite of heterogeneous biomarkers. The ability to identify individuals at risk for poor outcomes after trauma exposure by leveraging passively collected smartphone data in daily life could facilitate early identification of those at risk for developing APNSs. In turn, such individuals could be connected with appropriate health care services before their symptoms become chronic. More research is needed to determine the most effective preventive interventions after trauma exposure, particularly given the diverse range of potential negative outcomes. However, research has indicated that cognitive behavioral preventive interventions delivered in the early aftermath of trauma may be promising for reducing PTS and related symptoms [28]. Utilizing keystroke biomarkers could also assist in clinical research by identifying and recruiting individuals at risk for APNSs after trauma exposure. Furthermore, the ability to detect state changes in APNSs is particularly valuable. Pending further research, Figure 1 depicts an example of a clinical tool that could be developed to leverage state data from passively collected biomarkers to complement assessments conducted by providers during clinical visits. Ongoing passive data collection indicating state changes in APNSs may ultimately be informative for health care providers and researchers for several reasons. First, providers are often only able to assess changes between appointments, which may occur at long intervals. Second, APNSs are often difficult or impossible to assess “objectively.” Third, APNSs are challenging to report retrospectively with accuracy [53]. Data derived from keystroke biomarkers that capture trajectories of APNSs over time could enhance assessment and improve the ability to evaluate treatment efficacy.

### Strengths and Limitations

Results of this study should be considered in the context of its strengths and limitations. Strengths include the large sample size of diverse trauma survivors presenting to EDs who were followed prospectively after trauma exposure. Regarding limitations, first, only individuals who presented for emergency care after trauma exposure were included. Many trauma survivors do not seek emergency care, and it is unclear whether these findings generalize to those populations. Second, the majority of the sample consisted of MVC survivors. This is consistent with rates of trauma exposure in the United States, where MVC is one of the most common traumas experienced [1]. However, compared with other trauma types, MVCs may confer a relatively lower risk of developing APNSs [10]. Therefore, results may not generalize to survivors of all trauma types. Third, only Android users were included because iOS users could opt out of keystroke data collection. Although we did not detect differences in clinical symptoms between Android and iOS users, restricting the sample to Android users may affect generalizability. Notably, demographic differences were observed between iOS and Android users; however, these differences suggested that Android users represented a more racially and ethnically diverse sample, which may enhance generalizability. Relatedly, an important consideration in digital phenotyping research is the balance between patient privacy and the potential clinical utility of passively sensed smartphone data. Although substantially more research is needed before keystroke biomarkers can be implemented in clinical settings, further work is also required to ensure the development of secure and trustworthy apps that adequately safeguard private information. Even with such safeguards, some individuals may choose not to use these apps due to privacy concerns. Ethical considerations surrounding these data sources remain ongoing, and the balance of risks and benefits should be discussed with all patients and participants who may use such technologies. Fourth, participants were followed for 8 weeks after trauma exposure to assess APNS outcomes. Research indicates that by this point in the posttrauma period, most individuals with ongoing APNSs will continue to experience chronic symptoms unless they receive treatment [7]. However, future research may benefit from longer follow-up periods after a traumatic event.

### Conclusions

Up to 90% of Americans experience a traumatic event at some point in their lives [1], and many go on to develop chronic APNSs [2]. To improve recovery after trauma, it is critical to identify individuals at risk of developing these symptoms. Our findings among 1072 diverse trauma survivors presenting to EDs nationwide indicate the potential utility of passively collected smartphone keystroke data to identify both individuals at risk for developing chronic pain, reexperiencing, and mental fatigue symptoms, as well as state-level worsening or recovery of pain, sleep disturbance, hyperarousal, reexperiencing, somatic symptoms, and mental fatigue in the 8 weeks after trauma exposure. In general, slower typing and scrolling speeds were associated with higher symptom levels, with small- to medium-effect sizes. Keystroke data passively collected via smartphone use in daily life show promise and warrant further research to determine whether they may serve as useful biomarkers for identifying trauma survivors at risk for APNSs. Future research should continue to explore associations between these keystroke biomarkers and APNSs, and assess whether they can be leveraged to connect vulnerable trauma survivors to appropriate health care services, preventive interventions, or both. Overall, these results add to the literature across other conditions indicating that passively collected keystroke data may help identify individuals with neuropsychiatric symptoms or changes. However, to our knowledge, this is the first study to test whether keystroke biomarkers are useful in identifying individuals at risk for neuropsychiatric symptoms in the aftermath of trauma—a critical period during which preventive interventions could be deployed to reduce the long-term burden of trauma-related sequelae.

## Appendix

Multimedia Appendix 1 Additional analysis.

## Abbreviations

APNS: adverse posttraumatic neuropsychiatric symptom
AURORA: Advancing Understanding of Recovery After Trauma
ED: emergency department
MVC: motor vehicle collision
PTS: posttraumatic stress

## Data Availability

Data used in this manuscript are available through the National Institute of Mental Health (NIMH) Data Archive (NDA). The NDA collection for the AURORA (Advancing Understanding of Recovery After Trauma) Project can be found online [54].

## References

[1] Kilpatrick DG, Resnick HS, Milanak ME, Miller MW, Keyes KM, Friedman MJ (2013). National estimates of exposure to traumatic events and PTSD prevalence using DSM-IV and DSM-5 criteria. J Trauma Stress.

[2] McLean S, Ressler K, Koenen K, Neylan T, Germine L, Jovanovic T, Clifford Gari D, Zeng Donglin, An Xinming, Linnstaedt Sarah, Beaudoin Francesca, House Stacey, Bollen Kenneth A, Musey Paul, Hendry Phyllis, Jones Christopher W, Lewandowski Christopher, Swor Robert, Datner Elizabeth, Mohiuddin Kamran, Stevens Jennifer S, Storrow Alan, Kurz Michael Christopher, McGrath Meghan E, Fermann Gregory J, Hudak Lauren A, Gentile Nina, Chang Anna Marie, Peak David A, Pascual Jose L, Seamon Mark J, Sergot Paulina, Peacock W Frank, Diercks Deborah, Sanchez Leon D, Rathlev Niels, Domeier Robert, Haran John Patrick, Pearson Claire, Murty Vishnu P, Insel Thomas R, Dagum Paul, Onnela Jukka-Pekka, Bruce Steven E, Gaynes Bradley N, Joormann Jutta, Miller Mark W, Pietrzak Robert H, Buysse Daniel J, Pizzagalli Diego A, Rauch Scott L, Harte Steven E, Young Larry J, Barch Deanna M, Lebois Lauren A M, van Rooij Sanne J H, Luna Beatriz, Smoller Jordan W, Dougherty Robert F, Pace Thaddeus W W, Binder Elisabeth, Sheridan John F, Elliott James M, Basu Archana, Fromer Menachem, Parlikar Tushar, Zaslavsky Alan M, Kessler Ronald (2020). The AURORA Study: a longitudinal, multimodal library of brain biology and function after traumatic stress exposure. Mol Psychiatry.

[3] Momartin S, Silove D, Manicavasagar V, Steel Z (2004). Comorbidity of PTSD and depression: associations with trauma exposure, symptom severity and functional impairment in Bosnian refugees resettled in Australia. Journal of Affective Disorders.

[4] Dobie DJ, Kivlahan DR, Maynard C, Bush KR, Davis TM, Bradley KA (2004). Posttraumatic stress disorder in female veterans: association with self-reported health problems and functional impairment. Arch Intern Med.

[5] Clapp J, Beck J, Palyo S, Grant D (2008). An examination of the synergy of pain and PTSD on quality of life: additive or multiplicative effects?. Pain.

[6] Gormsen L, Rosenberg R, Bach FW, Jensen TS (2010). Depression, anxiety, health-related quality of life and pain in patients with chronic fibromyalgia and neuropathic pain. Eur J Pain.

[7] Rothbaum BO, Foa EB, Riggs DS, Murdock T, Walsh W (1992). A prospective examination of post-traumatic stress disorder in rape victims. J Trauma Stress.

[8] Bortsov A, Platts-Mills T F, Peak D, Jones J, Swor R, Domeier R, Lee D, Rathlev N, Hendry P, Fillingim R, McLean S (2013). Pain distribution and predictors of widespread pain in the immediate aftermath of motor vehicle collision. Eur J Pain.

[9] Vollmer Dahlke Deborah, Fair K, Hong YA, Beaudoin CE, Pulczinski J, Ory MG (2015). Apps seeking theories: results of a study on the use of health behavior change theories in cancer survivorship mobile apps. JMIR Mhealth Uhealth.

[10] Smith HL, Summers BJ, Dillon KH, Cougle JR (2016). Is worst-event trauma type related to PTSD symptom presentation and associated features?. J Anxiety Disord.

[11] Kessler RC, Aguilar-Gaxiola S, Alonso J, Benjet C, Bromet EJ, Cardoso G, Degenhardt L, de Girolamo G, Dinolova RV, Ferry F, Florescu S, Gureje O, Haro JM, Huang Y, Karam EG, Kawakami N, Lee S, Lepine J, Levinson D, Navarro-Mateu F, Pennell B, Piazza M, Posada-Villa J, Scott KM, Stein DJ, Ten Have M, Torres Y, Viana MC, Petukhova MV, Sampson NA, Zaslavsky AM, Koenen KC (2017). Trauma and PTSD in the WHO World Mental Health Surveys. Eur J Psychotraumatol.

[12] Holm LW, Carroll LJ, Cassidy JD, Skillgate E, Ahlbom A (2007). Widespread pain following whiplash-associated disorders: incidence, course, and risk factors. J Rheumatol.

[13] Berglund A, Alfredsson L, Jensen I, Cassidy J, Nygren (2001). The association between exposure to a rear-end collision and future health complaints. J Clin Epidemiol.

[14] Carroll LJ, Holm LW, Hogg-Johnson S, Côté P, Cassidy JD, Haldeman S, Nordin M, Hurwitz EL, Carragee EJ, van der Velde G, Peloso PM, Guzman J (2008). Course and prognostic factors for neck pain in whiplash-associated disorders (WAD). Spine.

[15] Cassidy JD, Carroll L, Côté P, Berglund A, Nygren (2003). Low back pain after traffic collisions. Spine.

[16] Côté P, Cassidy J D, Carroll L (2000). The factors associated with neck pain and its related disability in the Saskatchewan population. Spine (Phila Pa 1976).

[17] Hincapié Cesar A, Cassidy J, Côté Pierre, Carroll L, Guzmán Jaime (2010). Whiplash injury is more than neck pain: a population-based study of pain localization after traffic injury. J Occup Environ Med.

[18] Kasch H, Bach FW, Jensen TS (2001). Handicap after acute whiplash injury: a 1-year prospective study of risk factors. Neurology.

[19] Sterling M, Jull G, Vicenzino B, Kenardy J, Darnell R (2005). Physical and psychological factors predict outcome following whiplash injury. Pain.

[20] Pilet C, Galinski M, Lafont S (2023). Chronic pain prevalence two years after a road crash and its biopsychosocial risk factors - results from the ESPARR cohort. Journal of Transport & Health.

[21] Jones GT, Nicholl BI, McBeth J, Davies KA, Morriss RK, Dickens C, Macfarlane GJ (2011). Role of road traffic accidents and other traumatic events in the onset of chronic widespread pain: results from a population-based prospective study. Arthritis Care Res (Hoboken).

[22] Vollmer Dahlke Deborah, Fair K, Hong YA, Beaudoin CE, Pulczinski J, Ory MG (2015). Apps seeking theories: results of a study on the use of health behavior change theories in cancer survivorship mobile apps. JMIR Mhealth Uhealth.

[23] Short NA, Lechner M, McLean BS, Tungate AS, Black J, Buchanan JA, Reese R, Ho JD, Reed GD, Platt MA, Riviello RJ, Rossi CH, Nouhan PP, Phillips CA, Martin SL, Liberzon I, Rauch SAM, Bollen KA, Kessler RC, McLean SA (2021). Health care utilization by women sexual assault survivors after emergency care: results of a multisite prospective study. Depress Anxiety.

[24] Vizer L, Pierce J, Ji Y, Bucher MA, Liu M, Ungar L, Giorgi S, Xing Z, House SL, Beaudoin FL, Stevens JS, Neylan TC, Clifford GD, Jovanovic T, Linnstaedt SD, Zeng D, Germine LT, Bollen KA, Rauch SL, Haran JP, Storrow AB, Lewandowski C, Musey PI, Hendry PL, Sheikh S, Jones CW, Punches BE, Hudak LA, Pascual JL, Seamon MJ, Harris E, Pearson C, Peak DA, Merchant RC, Domeier RM, O'Neil Brian J, Sergot P, Sanchez LD, Bruce SE, Harte SE, Kessler RC, Koenen KC, McLean SA, An X (2025). Smartphone language features may help identify adverse post-traumatic neuropsychiatric sequelae and their trajectories. NPP Digit Psychiatry Neurosci.

[25] López Cristina M, Andrews AR, Chisolm AM, de Arellano MA, Saunders B, Kilpatrick DG (2017). Racial/ethnic differences in trauma exposure and mental health disorders in adolescents. Cultural Diversity & Ethnic Minority Psychology.

[26] Carbone JT, Holzer KJ, Vaughn MG (2019). Posttraumatic stress disorder among low-income adolescents experiencing family-neighborhood income disparities. J Trauma Stress.

[27] McLaughlin KA, Alvarez K, Fillbrunn M, Green JG, Jackson JS, Kessler RC, Sadikova E, Sampson NA, Vilsaint CL, Williams DR, Alegría M (2018). Racial/ethnic variation in trauma-related psychopathology in the United States: a population-based study. Psychol Med.

[28] Short NA, Morabito DM, Gilmore AK (2020). Secondary prevention for posttraumatic stress and related symptoms among women whohave experienced a recent sexual assault: a systematic review and meta-analysis. Depress Anxiety.

[29] Bryant RA, Moulds ML, Nixon RV (2003). Cognitive behaviour therapy of acute stress disorder: a four-year follow-up. Behav Res Ther.

[30] (2021). Mobile fact sheet. Pew Research Center.

[31] Vesel C, Rashidisabet H, Zulueta J, Stange J, Duffecy J, Hussain F, Piscitello Andrea, Bark John, Langenecker Scott A, Young Shannon, Mounts Erin, Omberg Larsson, Nelson Peter C, Moore Raeanne C, Koziol Dave, Bourne Keith, Bennett Casey C, Ajilore Olusola, Demos Alexander P, Leow Alex (2020). Effects of mood and aging on keystroke dynamics metadata and their diurnal patterns in a large open-science sample: a BiAffect iOS study. J Am Med Inform Assoc.

[32] Zulueta J, Piscitello A, Rasic M, Easter R, Babu P, Langenecker SA, McInnis M, Ajilore O, Nelson PC, Ryan K, Leow A (2018). Predicting mood disturbance severity with mobile phone keystroke metadata: a biaffect digital phenotyping study. J Med Internet Res.

[33] Mastoras R, Iakovakis D, Hadjidimitriou S, Charisis V, Kassie S, Alsaadi T, Khandoker A, Hadjileontiadis LJ (2019). Touchscreen typing pattern analysis for remote detection of the depressive tendency. Sci Rep.

[34] Cao B, Zheng L, Zhang C, Yu P, Piscitello A, Zulueta J (2017). Deepmood: modeling mobile phone typing dynamics for mood detection. Proceedings of the 23rd ACM SIGKDD International Conference on Knowledge Discovery and Data Mining.

[35] Ajilore O, Bark JS, Demos AP, Zulueta J, Stange J, Duffecy J, Hussain F, Langenecker SA, Nelson P, Ryan K, McInnis MG, Leow A (2025). Assessment of cognitive function in bipolar disorder with passive smartphone keystroke metadata: a BiAffect digital phenotyping study. Front Psychiatry.

[36] Ning E, Estabrook R, Tulabandhula T, Zulueta J, Ross MK, Kabir S, Hussain F, Langenecker SA, Ajilore O, Leow A, Demos AP (2025). Predicting cognitive functioning in mood disorders through smartphone typing dynamics. Journal of Psychopathology and Clinical Science.

[37] Schultz L, Murphy M, Donegan M, Knights J, Baker J, Thompson M, Waters Andrew J, Roy Michael, Gray Joshua C (2025). Evaluating the acceptability and feasibility of collecting passive smartphone data to estimate psychological functioning in U.S. Service Members and Veterans: a pilot study. Mil Med.

[38] Matias P, Afonseca P, Nunes F, Henriques A, Gonçalves C, Rodrigues A (2025). Smartphone keyboard typing for rheumatic disease identification: a machine learning approach.

[39] Zhu N, Sarawgi A, Bühner Markus, Baumeister H, Garatva P, Ehring T, Terhorst Y (2025). The relation between passively collected data and PTSD: a systematic review and meta-analysis. NPJ Digit Med.

[40] Insel T, Cuthbert B, Garvey M, Heinssen R, Pine D, Quinn K, Sanislow Charles, Wang Philip (2010). Research domain criteria (RDoC): toward a new classification framework for research on mental disorders. Am J Psychiatry.

[41] (2025). APA Style Journal Article Reporting Standards (APA Style JARS). American Psychological Association (APA).

[42] Bijur P, Latimer C, Gallagher E (2003). Validation of a verbally administered numerical rating scale of acute pain for use in the emergency department. Acad Emergency Med.

[43] Cella D, Riley W, Stone A, Rothrock N, Reeve B, Yount S, Amtmann D, Bode R, Buysse D, Choi S, Cook K, Devellis Robert, DeWalt D, Fries JF, Gershon R, Hahn EA, Lai J, Pilkonis P, Revicki D, Rose M, Weinfurt K, Hays R, PROMIS Cooperative Group (2010). The Patient-Reported Outcomes Measurement Information System (PROMIS) developed and tested its first wave of adult self-reported health outcome item banks: 2005-2008. J Clin Epidemiol.

[44] Morin C, Belleville G, Bélanger Lynda, Ivers H (2011). The Insomnia Severity Index: psychometric indicators to detect insomnia cases and evaluate treatment response. Sleep.

[45] Weathers FW, Bovin MJ, Lee DJ, Sloan DM, Schnurr PP, Kaloupek DG, Keane TM, Marx BP (2018). The Clinician-Administered PTSD Scale for DSM–5 (CAPS-5): development and initial psychometric evaluation in military veterans. Psychological Assessment.

[46] Auvergne L, Bortsov AV, Ulirsch JC, Peak DA, Jones JS, Swor RA, Domeier RM, Lee DC, Rathlev NK, Hendry PL, McLean SA (2016). Association of epidemiologic factors and genetic variants influencing hypothalamic-pituitary-adrenocortical axis function with postconcussive symptoms after minor motor vehicle collision. Psychosom Med.

[47] Blevins Christy A, Weathers Frank W, Davis Margaret T, Witte Tracy K, Domino Jessica L (2015). The Posttraumatic Stress Disorder Checklist for DSM-5 (PCL-5): development and initial psychometric evaluation. J Trauma Stress.

[48] Hu L, Bentler PM (1999). Cutoff criteria for fit indexes in covariance structure analysis: conventional criteria versus new alternatives. Structural Equation Modeling: A Multidisciplinary Journal.

[49] Niska Richard, Bhuiya Farida, Xu Jianmin (2010). National Hospital Ambulatory Medical Care Survey: 2007 emergency department summary. Natl Health Stat Report.

[50] Lam K, Meijer K, Loonstra F, Coerver E, Twose J, Redeman E, Moraal B, Barkhof F, de Groot V, Uitdehaag B, Killestein J (2020). Real-world keystroke dynamics are a potentially valid biomarker for clinical disability in multiple sclerosis. Mult Scler.

[51] Walter KH, Palmieri PA, Gunstad J (2010). More than symptom reduction: changes in executive function over the course of PTSD treatment. Journal of Traumatic Stress.

[52] Simmons A, Strigo I, Matthews S, Paulus M, Stein M (2009). Initial evidence of a failure to activate right anterior insula during affective set shifting in posttraumatic stress disorder. Psychosom Med.

[53] Nahleen S, Nixon RD, Takarangi MK (2019). Current PTSD symptomatology distorts memory for past symptoms. Psychiatry Res.

[54] National Institutes of Health.

