# Supplementary material for: Smartphone Keystroke Biomarkers as Predictors of Adverse Neuropsychiatric Sequelae After Trauma in Trauma Survivors: Prospective Observational Cohort Study
Source: J Med Internet Res. 2026 Jun 1;28:e73771. doi: 10.2196/73771 (PMC13270168; doi:10.2196/73771)
Supplement: Multimedia Appendix 1 [file jmir_v28i1e73771_app1.docx]

**Supplemental Materials**

**Table 1.** Comparison of adverse posttraumatic neuropsychiatric symptoms between Android and iOS using trauma survivors participating in an observational cohort study

**Table 2.** Demographic and trauma characteristics comparison between Android and iOS using trauma survivors participating in an observational cohort study

**Table 3.** Symptom latent constructs indicator variable questions for adverse posttraumatic neuropsychiatric sequelae from Flash Surveys administered to trauma survivors participating in an observational cohort study.

**Table 4.** Trait keystroke biomarkers captured via mobile application and associations with adverse posttraumatic neuropsychiatric sequelae among trauma survivors participating in an observational cohort study.

**Table 5.** State keystroke biomarkers captured via mobile application and associations with adverse posttraumatic neuropsychiatric sequelae among trauma survivors participating in an observational cohort study.

**Table 6.** Prediction of worsening adverse posttraumatic neuropsychiatric symptoms using state keystroke biomarkers captured via mobile application among trauma survivors participating in an observational cohort study.

**Table 7.** Prediction of recovery from adverse posttraumatic neuropsychiatric symptoms using state keystroke biomarkers captured via mobile application among trauma survivors participating in an observational cohort study.

**Table 8.** Correlation of main adverse posttraumatic neuropsychiatric outcomes after trauma exposure at different timepoints with completion rate of four main assessments (to explore the impact of pre- and post-traumatic symptoms on missing data.

**Figure 1.** Data flow pathways for self-report and Keystroke data.

**Supplemental Figure 2.** Visual depiction of study flow and missing data from trauma survivors participating in an observational cohort study.

**Sample Analysis Code**

- R example code

**Supplementary Table 1.**

Comparison of adverse posttraumatic neuropsychiatric symptoms between Android and iOS using trauma survivors participating in an observational cohort study

|  | | | |
| --- | --- | --- | --- |
|  | **Android Users** | **IOS Users** | **P-value** |
| Anxiety | 818  1.39 (0.94) | 789  1.33 (0.93) | 0.177 |
| Avoidance | 808  1.89 (0.96) | 805  1.80 (0.94) | 0.081 |
| Depression | 818  1.39 (0.98) | 789  1.37 (0.98) | 0.614 |
| Nightmare | 805  1.17 (1.00) | 831  1.08 (0.96) | 0.045 |
| Hyperarousal | 818  2.29 (0.86) | 789  2.24 (0.86) | 0.208 |
| Reexperiencing | 808  2.10 (0.99) | 805  1.98 (1.00) | 0.014 |
| Sleep Disturbance | **805**  **1.97 (0.79)** | **831**  **1.83 (0.79)** | **< 0.001** |
| Concentration/Thinking/Fatigue | 794  5.25 (1.84) | 842  6.26 (1.76) | 0.99 |
| Pain | **768**  **6.28 (1.88)** | **825**  **6.05 (1.98)** | **0.019** |
| Somatic Symptoms | 794  4.81 (2.17) | 842  4.69 (2.21) | 0.234 |

*Note.* **Bolded** figures represent statistically significant differences. Given the large sample size, statistical significance was not unexpected, so we reviewed clinical significance of differences. Differences were not considered clinically significant. Clinically significant changes in pain are typically considered to be at least a 2-point change on the pain numeric rating scale (NRS).^1^ For sleep disturbance, there was not a specific cut-off for clinically significant change in the literature given we used an abbreviated measure. However, the items were rated on a 0-4 point scale. Both groups demonstrated an average of 2 (sometimes), and only differed by 0.14, suggesting minimal differences between groups.

**Supplemental Table 2.**

| Demographic and trauma characteristics comparison between Android and iOS using trauma survivors participating in an observational cohort study. | | | |
| --- | --- | --- | --- |
| **Characteristic** | **ANDROID Users**  **(N = 1,072)** | **IOS Users**  **(N = 1,104)** | **P-value** |
| Age (y), Mean (SD) | 39.4 (12.6) | 32.0 (12.5) | < 0.001 |
| Female, n (%) | 616 (57.5) | 738 (66.8) | < 0.001 |
| Race, n (%) |  |  | 0.003 |
| Hispanic | 114 (10.6) | 137 (12.4) |  |
| Non-Hispanic White | 353 (32.9) | 416 (37.7) |  |
| Non-Hispanic Black | 565 (52.7) | 497 (45.0) |  |
| Non-Hispanic Other | 34 (3.2) | 50 (4.5) |  |
| Education Status, n (%) |  |  | < 0.001 |
| High school or less | 152 (14.2) | 86 (7.8) |  |
| Some college | 727 (67.8) | 728 (65.9) |  |
| College or more | 189 (17.6) | 287 (26.0) |  |
| Marital Status, n (%) |  |  | < 0.001 |
| Married | 245 (22.9) | 205 (18.6) |  |
| Separated, divorced, widowed, or annulled | 238 (22.2) | 138 (12.5) |  |
| Never been married | 578 (53.9) | 758 (68.7) |  |
| Trauma Type, n (%) |  |  | 0.005 |
| Motor vehicle collision | 772 (72.0) | 865 (78.4) |  |
| Physical assault | 118 (11.0) | 77 (7.0) |  |
| Sexual assault | 4 (0.4) | 10 (0.9) |  |
| Fall | 83 (7.8) | 68 (6.1) |  |
| Non-motorized collision | 21 (2.0) | 22 (2.0) |  |
| Animal-related | 20 (1.9) | 26 (2.4) |  |
| Other (including poisoning, burns, mass  trauma exposure) | 54 (5.1) | 36 (3.3) |  |

**Supplementary Table 3.**

Symptom latent constructs indicator variable questions for adverse posttraumatic neuropsychiatric sequelae from Flash Surveys administered to trauma survivors participating in an observational cohort study.

| **Latent Constructs** | **Timepoints (Days)**  **(first 6 months)** | **Questions** | **Original Measure** | **Response options** |
| --- | --- | --- | --- | --- |
| **Pain** | 1,9 ,21,31,43,53, 67,77,105,147,196 | 1. How would you rate your pain in the past 24 hours at its worst? 2. How would you rate your pain in the past 24 hours on average? | Numeric Pain Rating Scale (NRS)^2^ | No pain Severe pain  0 1 2 3 4 5 6 7 8 9 10 |
| **Loss** | 5,19,29,39,51,61,  75,91,133,175 | 1. Over the past 24 hours, how often did you feel down on yourself, no good, or worthless? 2. Over the past 24 hours, how often did you feel sad depressed, or empty? 3. Over the past 24 hours, how often did you have trouble experiencing positive feelings? (for example, being unable to feel happiness or having loving feelings for people close to you) | PROMIS Depression Short Form 8b^3^ | Never Rarely Sometimes Often Very often  [0] [1] [2] [3] [4] |
| **Sleep Disturbance** | 3,15,25,35,47,57,  71,81,119,161 | 1. Over the last few nights, how much of a problem have you had falling asleep? 2. Over the last few nights, how much of a problem have you had staying asleep all night? 3. Over the last few nights, how much of a problem have you had waking up too early in the morning? | PROMIS Sleep Disturbance – Short Form^4^ | None A little Some A lot Extremely  [0] [1] [2] [3] [4] |
| **Nightmares** | 3,15,25,35,47,57,  71,81,119,161 | 1. Over the last few nights, how much of a problem have you had with nightmares or bad dreams about the event? 2. Over the last few nights, how much of a problem have you had with nightmares or bad dreams about other things? 3. Over the last few nights, how much of a problem have you had with panic attacks during the night? | Clinician Administered PTSD Scale (CAPS)-IV^5^ | None A little Some A lot Extremely  [0] [1] [2] [3] [4] |
| **Anxiety** | 5,19,29,39,51,61,  75,91,133,175 | 1. Over the past 24 hours, how often did you have severe anxiety or panic? 2. Over the past 24 hours, how often did you feel very nervous, worried, or anxious? | PROMIS Anxiety Bank^6^ | Never Rarely Sometimes Often Very often  [0] [1] [2] [3] [4] |
| **Hyperarousal** | 5,19,29,39,51,61,  75,91,133,175 | 1. Over the past 24 hours, how often were you “superalert” or watchful, or on guard? 2. Over the past 24 hours, how often did you feel jumpy or easily startled? | PTSD Checklist for DSM-5 (PCL-5)^7^ | Never Rarely Sometimes Often Very often  [0] [1] [2] [3] [4] |
| **Avoidance** | 4,17,27,37,49,59,  73,83,126,168 | 1. Over the past 24 hours, how often did you avoid memories, thoughts, or feelings related to the event? 2. Over the past 24 hours, how often did you avoid external reminders of the event? (e.g., people, places, conversations, or activities) | PCL-5^7^ | Never Rarely Sometimes Often Very often  [0] [1] [2] [3] [4] |
| **Re-experiencing** | 4,17,27,37,49,59,  73,83,126,168 | 1. Over the past 24 hours, how often did you have repeated, disturbing, and unwanted memories of the event? 2. Over the past 24 hours, how often did you feel very upset when something reminded you of the event? 3. Over the past 24 hours, how often did you have strong physical reactions when something reminded you of the event, like heart pounding, trouble breathing, or sweating? | PCL-5^7^ | Never Rarely Sometimes Often Very often  [0] [1] [2] [3] [4] |
| **Somatic Symptoms** | 2,11,23,33,45,55,69,  79,112,154,203 | 1. Over the past 24 hours, how much of a problem have you had with headaches? 2. Over the past 24 hours, how much of a problem have you had with dizziness? 3. Over the past 24 hours, how much of a problem have you had with nausea? | Rivermead Post-Concussive Questionnaire (RPQ)-12^8^ | No problem Severe problem  0 1 2 3 4 5 6 7 8 9 10 |
| **Mental Fatigue** | 2,11,23,33,45,55,69,  79,112,154,203 | 1. Over the past 24 hours, how much of a problem have you had with fatigue? 2. Over the past 24 hours, how much of a problem have you had concentrating? 3. Over the past 24 hours, how much of a problem have you had taking longer to think? | RPQ-12^8^ | No problem Severe problem  0 1 2 3 4 5 6 7 8 9 10 |

**Supplementary Table 4.**

Trait keystroke biomarkers captured via mobile application and associations with adverse posttraumatic neuropsychiatric sequelae among trauma survivors participating in an observational cohort study.

| **Construct** | **Theme** | **Type** | **Signal Processing** | **Correlation**  **(95% CI)** | **Bonferroni adjusted *p*-value** |
| --- | --- | --- | --- | --- | --- |
| Pain | Typing speed | Two characters in a row | Standard deviation | 0.23 (0.14, 0.31) | <0.001 |
|  | Typing speed | Two characters in a row | Mean log | 0.25 (0.16, 0.34) | <0.001 |
|  | Typing speed | Two characters in a row | 50^th^ percentile | 0.25 (0.17, 0.34) | <0.001 |
|  | Typing speed | Two characters in a row | 80^th^ percentile | 0.25 (0.17, 0.34) | <0.001 |
|  | Typing speed | Two characters in a row | 90^th^ percentile | 0.25 (0.16, 0.33) | <0.001 |
|  | Typing speed | Two characters in a row | Total (sum) power | -0.26 (-0.34, -0.18) | <0.001 |
|  | Typing speed | Two characters in a row | Mean frequency | -0.26 (-0.34, -0.17) | <0.001 |
|  | Typing speed | Two characters in a row | 1^st^ spectral moment | -0.26 (-0.34, -0.17) | <0.001 |
|  | Typing speed | Two characters in a row | 2^nd^ spectral moment | -0.26 (-0.34, -0.17) | <0.001 |
|  | Typing speed | Two characters in a row | 3^rd^ spectral moment | -0.26 (-0.34, -0.17) | <0.001 |
|  | Deletions | Two deletes in a row | Mean | 0.20 (0.11, 0.28) | <0.001 |
|  | Deletions | Two deletes in a row | Mean log | 0.20 (0.11, 0.29) | <0.001 |
|  | Deletions | Two deletes in a row | Total (sum) power | -0.20 (-0.28, -0.11) | <0.001 |
|  | Deletions | Two deletes in a row | 1^st^ spectral moment | -0.19 (-0.28, -0.10) | <0.001 |
|  | Typing speed | Two characters | Mean log | 0.25 (0.17, 0.34) | <0.001 |
|  | Typing speed | Two characters | 10^th^ percentile | 0.25 (0.16, 0.33) | <0.001 |
|  | Typing speed | Two characters | 20^th^ percentile | 0.25 (0.16, 0.33) | <0.001 |
|  | Typing speed | Two characters | 50^th^ percentile | 0.25 (0.17, 0.34) | <0.001 |
|  | Typing speed | Two characters | 80^th^ percentile | 0.24 (0.16, 0.33) | <0.001 |
|  | Typing speed | Two characters | 90^th^ percentile | 0.24 (0.15, 0.33) | <0.001 |
|  | Typing speed | Two characters | Total (sum) power | -0.26 (-0.34, -0.17) | <0.001 |
|  | Typing speed | Two characters | Mean frequency | -0.26 (-0.34, -0.18) | <0.001 |
|  | Typing speed | Two characters | 1^st^ spectral moment | -0.26 (-0.34, -0.17) | <0.001 |
|  | Typing speed | Two characters | 2^nd^ spectral moment | -0.26 (-0.34, -0.17) | <0.001 |
|  | Typing speed | Two characters | 3^rd^ spectral moment | -0.26 (-0.34, -0.17) | <0.001 |
|  | Typing speed | Two characters | Mean log | 0.25 (0.16, 0.33) | <0.001 |
|  | Typing speed | Two characters | 50^th^ percentile | 0.26 (0.17, 0.34) | <0.001 |
|  | Typing speed | Two characters | 80^th^ percentile | 0.25 (0.16, 0.33) | <0.001 |
|  | Typing speed | Two characters | 90^th^ percentile | 0.24 (0.15, 0.32) | <0.001 |
|  | Typing speed | Two characters | Total (sum) power | -0.26 (-0.34, -0.17) | <0.001 |
|  | Typing speed | Two characters | 1^st^ spectral moment | -0.26 (-0.34, -0.17) | <0.001 |
|  | Typing speed | Two characters | 2^nd^ spectral moment | -0.26 (-0.34, -0.17) | <0.001 |
|  | Typing speed | Two characters | 3^rd^ spectral moment | -0.26 (-0.34, -0.17) | <0.001 |
|  | Typing speed | ≤ 3 characters in a row | Mean log | 0.25 (0.16, 0.33) | ≤0.001 |
|  | Typing speed | ≤ 3 characters in a row | 50^th^ percentile | 0.25 (0.17, 0.34) | <0.001 |
|  | Typing speed | ≤ 3 characters in a row | 80^th^ percentile | 0.25 (0.16, 0.33) | <0.001 |
|  | Typing speed | ≤ 3 characters in a row | 90^th^ percentile | 0.25 (0.16, 0.33) | <0.001 |
|  | Typing speed | ≤ 3 characters in a row | Total (sum) power | -0.26 (-0.34, -0.17) | <0.001 |
|  | Typing speed | ≤ 3 characters in a row | 1^st^ spectral moment | -0.26 (-0.34, -0.17) | <0.001 |
|  | Typing speed | ≤ 3 characters in a row | 2^nd^ spectral moment | -0.25 (-0.34, -0.17) | <0.001 |
|  | Typing speed | ≤ 3 characters in a row | 3^rd^ spectral moment | -0.25 (-0.34, -0.17) | <0.001 |
|  | Typing speed | > 3 characters in a row | Mean | 0.24 (0.16, 0.33) | <0.001 |
|  | Typing speed | > 3 characters in a row | Mean log | 0.26 (0.17, 0.34) | <0.001 |
|  | Typing speed | > 3 characters in a row | 50^th^ percentile | 0.25 (0.17, 0.34) | <0.001 |
|  | Typing speed | > 3 characters in a row | 80^th^ percentile | 0.26 (0.17, 0.34) | <0.001 |
|  | Typing speed | > 3 characters in a row | 90^th^ percentile | 0.25 (0.17, 0.34) | <0.001 |
|  | Typing speed | > 3 characters in a row | Total (sum) power | -0.26 (-0.35, -0.18) | <0.001 |
|  | Typing speed | > 3 characters in a row | 1^st^ spectral moment | -0.26 (-0.35, -0.18) | <0.001 |
|  | Typing speed | > 3 characters in a row | 2^nd^ spectral moment | -0.26 (-0.34, -0.18) | <0.001 |
|  | Typing speed | > 3 characters in a row | 3^rd^ spectral moment | -0.26 (-0.34, -0.17) | <0.001 |
| Hyperarousal | Change of operations | Delete to type | Mean power | -0.17 (-0.25, -0.08) | <0.001 |
| Avoidance | Change of operations | Delete to type | Mean power | -0.17 (-0.25, -0.09) | <0.001 |
| Re-experiencing | Typing speed | Character to space | Mean power | -0.17 (-0.25, -0.09) | <0.001 |
|  | Change of operations | Delete to type | Mean power | -0.18 (-0.26, -0.10) | <0.001 |
|  | Typing speed | ≤ 4 characters then space | Mean power | -0.18 (-0.26, -0.09) | <0.001 |
|  | Typing speed | The second set of at least 5 characters in a row | Mean power | -0.17 (-0.25, -0.08) | <0.001 |
| Somatic Symptoms | Deletions | Two deletes in a row | Mean | 0.17 (0.09, 0.25) | <0.001 |
|  | Deletions | Two deletes in a row | Mean log | 0.17 (0.09, 0.25) | <0.001 |
|  | Deletions | Two deletes in a row | 50^th^ percentile | 0.18 (0.09, 0.26) | <0.001 |
|  | Deletions | Two deletes in a row | Total (sum) power | -0.17 (-0.25, -0.08) | <0.001 |
|  | Typing speed | > 3 characters in a row | Standard deviation | 0.18 (0.09, 0.26) | <0.001 |
| Difficulty with concentration/thinking/fatigue | Scroll | Scroll then click | Mean | 0.17 (0.08, 0.25) | <0.001 |
|  | Scroll | Scroll then click | Mean log | 0.17 (0.09, 0.25) | <0.001 |
|  | Scroll | Scroll then click | Total (sum) power | -0.17 (-0.25, -0.09) | <0.001 |
|  | Scroll | Scroll then click | 1^st^ spectral moment | -0.17 (-0.25, -0.09) | <0.001 |
|  | Scroll | Scroll then click | 2^nd^ spectral moment | -0.17 (-0.25, -0.09) | <0.001 |
|  | Scroll | Scroll then click | 3^rd^ spectral moment | -0.17 (-0.26, -0.09) | <0.001 |

**Supplementary Table 5.**

State keystroke biomarkers captured via mobile application and associations with adverse posttraumatic neuropsychiatric sequelae among trauma survivors participating in an observational cohort study.

| **Construct** | **Theme** | **Type** | **Signal Processing** | **Correlation**  **(95% CI)** | **Bonferroni adjusted *p*-value** |
| --- | --- | --- | --- | --- | --- |
| Pain | Typing speed | Two characters in a row | Total (sum) power | -0.07 (-0.11, -0.04) | <0.001 |
|  | Typing speed | Two characters in a row | Mean frequency | -0.09 (-0.12, -0.06) | <0.001 |
|  | Typing speed | Two characters in a row | 1^st^ spectral moment | -0.08 (-0.11, -0.05) | <0.001 |
|  | Typing speed | Two characters in a row | 2^nd^ spectral moment | -0.08 (-0.12, -0.05) | <0.001 |
|  | Typing speed | Two characters in a row | 3^rd^ spectral moment | -0.09 (-0.12, -0.05) | <0.001 |
|  | Typing speed | Two characters | Total (sum) power | -0.07 (-0.1, -0.04) | <0.001 |
|  | Typing speed | Two characters | Mean frequency | -0.09 (-0.12, -0.06) | <0.001 |
|  | Typing speed | Two characters | 1^st^ spectral moment | -0.08 (-0.11, -0.05) | <0.001 |
|  | Typing speed | Two characters | 2^nd^ spectral moment | -0.08 (-0.11, -0.05) | <0.001 |
|  | Typing speed | Two characters | 3^rd^ spectral moment | -0.08 (-0.12, -0.05) | <0.001 |
|  | Typing speed | Two characters | Total (sum) power | -0.07 (-0.10, -0.04) | <0.001 |
|  | Typing speed | Two characters | Mean frequency | -0.09 (-0.12, -0.05) | <0.001 |
|  | Typing speed | Two characters | 1^st^ spectral moment | -0.08 (-0.11, -0.05) | <0.001 |
|  | Typing speed | Two characters | 2^nd^ spectral moment | -0.08 (-0.12, -0.05) | <0.001 |
|  | Typing speed | Two characters | 3^rd^ spectral moment | -0.09 (-0.12, -0.05) | <0.001 |
|  | Typing speed | Consecutive typing events | Mean log | 0.08 (0.04, 0.11) | <0.001 |
|  | Typing speed | Consecutive typing events | 20^th^ percentile | 0.07 (0.04, 0.10) | <0.001 |
|  | Typing speed | Consecutive typing events | 50^th^ percentile | 0.07 (0.04, 0.11) | <0.001 |
|  | Typing speed | Consecutive typing events | 80^th^ percentile | 0.07 (0.03, 0.10) | <0.001 |
|  | Typing speed | Consecutive typing events | 90^th^ percentile | 0.07 (0.03, 0.10) | <0.001 |
|  | Typing speed | Consecutive typing events | Total (sum) power | -0.09 (-0.12, -0.05) | <0.001 |
|  | Typing speed | Consecutive typing events | Median frequency | -0.08 (-0.12, -0.05) | <0.001 |
|  | Typing speed | Consecutive typing events | Mean frequency | -0.10 (-0.13, -0.07) | <0.001 |
|  | Typing speed | Consecutive typing events | 1^st^ spectral moment | -0.09 (-0.12, -0.06) | <0.001 |
|  | Typing speed | Consecutive typing events | 2^nd^ spectral moment | -0.09 (-0.13, -0.06) | <0.001 |
|  | Typing speed | Consecutive typing events | 3^rd^ spectral moment | -0.1 (-0.13, -0.06) | <0.001 |
|  | Scroll | Scroll twice to item | Maximum fractal length | -0.09 (-0.12, -0.06) | <0.001 |
|  | Typing speed | ≤ 3 characters in a row | Mean frequency | -0.07 (-0.100, -0.04) | <0.001 |
|  | Typing speed | > 3 characters in a row | Mean frequency | -0.07 (-0.11, -0.04) | <0.001 |
|  | Typing speed | > 3 characters in a row | 1^st^ spectral moment | -0.07 (-0.10, -0.04) | <0.001 |
|  | Typing speed | > 3 characters in a row | 2^nd^ spectral moment | -0.07 (-0.10, -0.04) | <0.001 |
|  | Typing speed | > 3 characters in a row | 3^rd^ spectral moment | -0.07 (-0.10, -0.04) | <0.001 |
| Depressive symptoms | Typing speed | The first set of at least 5 characters in a row | Maximum fractal length | 0.06 (0.03, 0.10) | <0.001 |
| Sleep Disturbance | Typing speed | Two characters in a row | Mean log | 0.07 (0.03, 0.10) | <0.001 |
|  | Typing speed | Two characters in a row | Total (sum) power | -0.07 (-0.10, -0.04) | <0.001 |
|  | Typing speed | Two characters in a row | Mean frequency | -0.07 (-0.10, -0.04) | <0.001 |
|  | Typing speed | Two characters in a row | 1^st^ spectral moment | -0.07 (-0.11, -0.04) | <0.001 |
|  | Typing speed | Two characters in a row | 2^nd^ spectral moment | -0.07 (-0.11, -0.04) | <0.001 |
|  | Typing speed | Two characters in a row | 3^rd^ spectral moment | -0.07 (-0.11, -0.04) | <0.001 |
|  | Typing speed | Two characters | Mean log | 0.07 (0.04, 0.10) | <0.001 |
|  | Typing speed | Two characters | 20^th^ percentile | 0.07 (0.04, 0.11) | <0.001 |
|  | Typing speed | Two characters | Total (sum) power | -0.07 (-0.10, -0.04) | <0.001 |
|  | Typing speed | Two characters | Mean frequency | -0.08 (-0.11, -0.04) | <0.001 |
|  | Typing speed | Two characters | 1^st^ spectral moment | -0.07 (-0.11, -0.04) | <0.001 |
|  | Typing speed | Two characters | 2^nd^ spectral moment | -0.07 (-0.11, -0.04) | <0.001 |
|  | Typing speed | Two characters | 3^rd^ spectral moment | -0.07 (-0.11, -0.04) | <0.001 |
|  | Typing speed | Two characters | Mean log | 0.07 (0.03, 0.10) | <0.001 |
|  | Typing speed | Two characters | 20^th^ percentile | 0.06 (0.03, 0.10) | <0.001 |
|  | Typing speed | Two characters | Total (sum) power | -0.07 (-0.10, -0.04) | <0.001 |
|  | Typing speed | Two characters | Mean frequency | -0.08 (-0.11, -0.04) | <0.001 |
|  | Typing speed | Two characters | 1^st^ spectral moment | -0.07 (-0.11, -0.04) | <0.001 |
|  | Typing speed | Two characters | 2^nd^ spectral moment | -0.08 (-0.11, -0.04) | <0.001 |
|  | Typing speed | Two characters | 3^rd^ spectral moment | -0.08 (-0.11, -0.04) | <0.001 |
|  | Typing speed | Consecutive typing events | Mean | 0.07 (0.04, 0.11) | <0.001 |
|  | Typing speed | Consecutive typing events | Mean log | 0.08 (0.05, 0.11) | <0.001 |
|  | Typing speed | Consecutive typing events | 20^th^ percentile | 0.07 (0.04, 0.11) | <0.001 |
|  | Typing speed | Consecutive typing events | 50^th^ percentile | 0.08 (0.04, 0.11) | <0.001 |
|  | Typing speed | Consecutive typing events | 80^th^ percentile | 0.07 (0.04, 0.10) | <0.001 |
|  | Typing speed | Consecutive typing events | 90^th^ percentile | 0.07 (0.03, 0.10) | <0.001 |
|  | Typing speed | Consecutive typing events | Total (sum) power | -0.08 (-0.12, -0.05) | <0.001 |
|  | Typing speed | Consecutive typing events | Mean frequency | -0.08 (-0.12, -0.05) | <0.001 |
|  | Typing speed | Consecutive typing events | 1^st^ spectral moment | -0.08 (-0.12, -0.05) | <0.001 |
|  | Typing speed | Consecutive typing events | 2^nd^ spectral moment | -0.08 (-0.12, -0.05) | <0.001 |
|  | Typing speed | Consecutive typing events | 3^rd^ spectral moment | -0.08 (-0.12, -0.05) | <0.001 |
|  | Typing speed | > 3 characters in a row | Mean | 0.08 (0.05, 0.11) | <0.001 |
|  | Typing speed | > 3 characters in a row | Mean log | 0.09 (0.05, 0.12) | <0.001 |
|  | Typing speed | > 3 characters in a row | 10^th^ percentile | 0.08 (0.05, 0.11) | <0.001 |
|  | Typing speed | > 3 characters in a row | 20^th^ percentile | 0.08 (0.05, 0.11) | <0.001 |
|  | Typing speed | > 3 characters in a row | 50^th^ percentile | 0.08 (0.05, 0.11) | <0.001 |
|  | Typing speed | > 3 characters in a row | 80^th^ percentile | 0.08 (0.05, 0.11) | <0.001 |
|  | Typing speed | > 3 characters in a row | 90^th^ percentile | 0.09 (0.05, 0.12) | <0.001 |
|  | Typing speed | > 3 characters in a row | Mean power | -0.09 (-0.12, -0.05) | <0.001 |
|  | Typing speed | > 3 characters in a row | Total (sum) power | -0.09 (-0.12, -0.06) | <0.001 |
|  | Typing speed | > 3 characters in a row | Mean frequency | -0.09 (-0.12, -0.06) | <0.001 |
|  | Typing speed | > 3 characters in a row | 1^st^ spectral moment | -0.09 (-0.13, -0.06) | <0.001 |
|  | Typing speed | > 3 characters in a row | 2^nd^ spectral moment | -0.09 (-0.13, -0.06) | <0.001 |
|  | Typing speed | > 3 characters in a row | 3^rd^ spectral moment | -0.09 (-0.13, -0.06) | <0.001 |
| Hyperarousal | Typing speed | Character to space | 1^st^ spectral moment | -0.07 (-0.11, -0.04) | <0.001 |
|  | Typing speed | Character to space | 2^nd^ spectral moment | -0.07 (-0.11, -0.04) | <0.001 |
|  | Typing speed | Character to space | 3^rd^ spectral moment | -0.07 (-0.11, -0.04) | <0.001 |
|  | Typing speed | Two characters in a row | Total (sum) power | -0.10 (-0.13, -0.06) | <0.001 |
|  | Typing speed | Two characters in a row | Mean frequency | -0.09 (-0.12, -0.06) | <0.001 |
|  | Typing speed | Two characters in a row | 1^st^ spectral moment | -0.10 (-0.13, -0.06) | <0.001 |
|  | Typing speed | Two characters in a row | 2^nd^ spectral moment | -0.1 (-0.13, -0.06) | <0.001 |
|  | Typing speed | Two characters in a row | 3^rd^ spectral moment | -0.09 (-0.13, -0.06) | <0.001 |
|  | Typing speed | Character to space | Total (sum) power | -0.09 (-0.12, -0.06) | <0.001 |
|  | Typing speed | Character to space | Mean frequency | -0.09 (-0.12, -0.05) | <0.001 |
|  | Typing speed | Character to space | 1^st^ spectral moment | -0.09 (-0.12, -0.06) | <0.001 |
|  | Typing speed | Character to space | 2^nd^ spectral moment | -0.09 (-0.12, -0.06) | <0.001 |
|  | Typing speed | Character to space | 3^rd^ spectral moment | -0.09 (-0.12, -0.06) | <0.001 |
|  | Typing speed | Two characters | 1^st^ spectral moment | -0.09 (-0.12, -0.06) | <0.001 |
|  | Typing speed | Two characters | 2^nd^ spectral moment | -0.09 (-0.12, -0.06) | <0.001 |
|  | Typing speed | Two characters | 3^rd^ spectral moment | -0.09 (-0.12, -0.06) | <0.001 |
|  | Typing speed | Consecutive typing events | 1^st^ spectral moment | -0.09 (-0.12, -0.06) | <0.001 |
|  | Typing speed | Consecutive typing events | 2^nd^ spectral moment | -0.09 (-0.12, -0.06) | <0.001 |
|  | Typing speed | Consecutive typing events | 3^rd^ spectral moment | -0.09 (-0.12, -0.05) | <0.001 |
|  | Typing speed | > 3 characters in a row | Mean log | 0.08 (0.05, 0.11) | <0.001 |
|  | Typing speed | > 3 characters in a row | 20^th^ percentile | 0.07 (0.04, 0.10) | <0.001 |
|  | Typing speed | > 3 characters in a row | Total (sum) power | -0.08 (-0.11, -0.05) | <0.001 |
|  | Typing speed | > 3 characters in a row | Mean frequency | -0.08 (-0.11, -0.05) | <0.001 |
|  | Typing speed | > 3 characters in a row | 1^st^ spectral moment | -0.08 (-0.11, -0.05) | <0.001 |
|  | Typing speed | > 3 characters in a row | 2^nd^ spectral moment | -0.08 (-0.11, -0.05) | <0.001 |
|  | Typing speed | > 3 characters in a row | 3^rd^ spectral moment | -0.08 (-0.11, -0.05) | <0.001 |
|  | Typing speed | The first set of at least 5 characters in a row | Standard deviation of the difference in differences | 0.06 (0.03, 0.10) | <0.001 |
|  | Typing speed | The first set of at least 5 characters in a row | Maximum fractal length | 0.07 (0.04, 0.11) | <0.001 |
|  | Typing speed | The first set of at least 5 characters in a row | Total (sum) power | -0.07 (-0.1, -0.03) | <0.001 |
|  | Typing speed | The first set of at least 5 characters in a row | 1^st^ spectral moment | -0.07 (-0.1, -0.03) | <0.001 |
|  | Typing speed | The first set of at least 5 characters in a row | 2^nd^ spectral moment | -0.07 (-0.1, -0.03) | <0.001 |
|  | Typing speed | The first set of at least 5 characters in a row | 3^rd^ spectral moment | -0.06 (-0.1, -0.03) | <0.001 |
|  | Typing speed | The second set of at least 5 characters in a row | Mean | 0.07 (0.04, 0.11) | <0.001 |
|  | Typing speed | The second set of at least 5 characters in a row | Mean log | 0.08 (0.05, 0.11) | <0.001 |
|  | Typing speed | The second set of at least 5 characters in a row | 10^th^ percentile | 0.07 (0.04, 0.11) | <0.001 |
|  | Typing speed | The second set of at least 5 characters in a row | 20^th^ percentile | 0.07 (0.04, 0.10) | <0.001 |
|  | Typing speed | The second set of at least 5 characters in a row | 50^th^ percentile | 0.07 (0.04, 0.11) | <0.001 |
|  | Typing speed | The second set of at least 5 characters in a row | 80^th^ percentile | 0.08 (0.04, 0.11) | <0.001 |
|  | Typing speed | The second set of at least 5 characters in a row | Mean power | -0.08 (-0.12, -0.05) | <0.001 |
|  | Typing speed | The second set of at least 5 characters in a row | Total (sum) power | -0.08 (-0.11, -0.04) | <0.001 |
|  | Typing speed | The second set of at least 5 characters in a row | Mean frequency | -0.07 (-0.10, -0.04) | <0.001 |
|  | Typing speed | The second set of at least 5 characters in a row | 1^st^ spectral moment | -0.08 (-0.11, -0.04) | <0.001 |
|  | Typing speed | The second set of at least 5 characters in a row | 2^nd^ spectral moment | -0.08 (-0.11, -0.04) | <0.001 |
|  | Typing speed | The second set of at least 5 characters in a row | 3^rd^ spectral moment | -0.07 (-0.11, -0.04) | <0.001 |
| Re-experiencing | Typing speed | Character to space | Mean log | 0.07 (0.03, 0.10) | <0.001 |
|  | Typing speed | Character to space | 10^th^ percentile | 0.07 (0.03, 0.10) | <0.001 |
|  | Typing speed | Character to space | 20^th^ percentile | 0.06 (0.03, 0.10) | <0.001 |
|  | Typing speed | Character to space | 90^th^ percentile | 0.07 (0.04, 0.10) | <0.001 |
|  | Typing speed | Character to space | Total (sum) power | -0.07 (-0.11, -0.04) | <0.001 |
|  | Typing speed | Character to space | Mean frequency | -0.08 (-0.11, -0.05) | <0.001 |
|  | Typing speed | Character to space | 1^st^ spectral moment | -0.08 (-0.11, -0.04) | <0.001 |
|  | Typing speed | Character to space | 2^nd^ spectral moment | -0.08 (-0.11, -0.04) | <0.001 |
|  | Typing speed | Character to space | 3^rd^ spectral moment | -0.07 (-0.11, -0.04) | <0.001 |
|  | Typing speed | Character to space | 10^th^ percentile | 0.07 (0.03, 0.10) | <0.001 |
|  | Typing speed | Character to space | Total (sum) power | -0.07 (-0.10, -0.03) | <0.001 |
|  | Typing speed | Character to space | Median frequency | -0.07 (-0.11, -0.04) | <0.001 |
|  | Typing speed | Character to space | Mean frequency | -0.08 (-0.11, -0.05) | <0.001 |
|  | Typing speed | Character to space | 1^st^ spectral moment | -0.07 (-0.10, -0.04) | <0.001 |
|  | Typing speed | Character to space | 2^nd^ spectral moment | -0.07 (-0.10, -0.04) | <0.001 |
|  | Typing speed | Character to space | 3^rd^ spectral moment | -0.07 (-0.10, -0.04) | <0.001 |
|  | Typing speed | Two characters | Mean frequency | -0.09 (-0.12, -0.06) | <0.001 |
|  | Typing speed | Consecutive typing events | Mean log | 0.07 (0.04, 0.10) | <0.001 |
|  | Typing speed | Consecutive typing events | Total (sum) power | -0.08 (-0.11, -0.05) | <0.001 |
|  | Typing speed | Consecutive typing events | Mean frequency | -0.08 (-0.12, -0.05) | <0.001 |
|  | Typing speed | Consecutive typing events | 1^st^ spectral moment | -0.08 (-0.12, -0.05) | <0.001 |
|  | Typing speed | Consecutive typing events | 2^nd^ spectral moment | -0.09 (-0.12, -0.05) | <0.001 |
|  | Typing speed | Consecutive typing events | 3^rd^ spectral moment | -0.09 (-0.12, -0.05) | <0.001 |
|  | Typing speed | ≤ 3 characters in a row | Total (sum) power | -0.08 (-0.11, -0.04) | <0.001 |
|  | Typing speed | ≤ 4 characters then space | Mean frequency | -0.07 (-0.10, -0.04) | <0.001 |
|  | Typing speed | ≤ 4 characters then space | 1^st^ spectral moment | -0.06 (-0.10, -0.03) | <0.001 |
|  | Typing speed | ≤ 4 characters then space | 2^nd^ spectral moment | -0.07 (-0.10, -0.03) | <0.001 |
|  | Typing speed | ≤ 4 characters then space | 3^rd^ spectral moment | -0.07 (-0.10, -0.03) | <0.001 |
| Somatic Symptoms | Typing speed | Two characters in a row | 80^th^ percentile | 0.06 (0.03, 0.09) | <0.001 |
|  | Typing speed | Two characters in a row | 90^th^ percentile | 0.07 (0.04, 0.10) | <0.001 |
|  | Typing speed | Two characters in a row | Total (sum) power | -0.08 (-0.11, -0.04) | <0.001 |
|  | Typing speed | Two characters in a row | Median frequency | -0.08 (-0.11, -0.05) | <0.001 |
|  | Typing speed | Two characters in a row | Mean frequency | -0.08 (-0.11, -0.05) | <0.001 |
|  | Typing speed | Two characters in a row | 1^st^ spectral moment | -0.08 (-0.11, -0.05) | <0.001 |
|  | Typing speed | Two characters in a row | 2^nd^ spectral moment | -0.08 (-0.12, -0.05) | <0.001 |
|  | Typing speed | Two characters in a row | 3^rd^ spectral moment | -0.08 (-0.12, -0.05) | <0.001 |
|  | Typing speed | Two characters | Total (sum) power | -0.07 (-0.10, -0.04) | <0.001 |
|  | Typing speed | Two characters | Median frequency | -0.07 (-0.11, -0.04) | <0.001 |
|  | Typing speed | Two characters | Mean frequency | -0.08 (-0.11, -0.05) | <0.001 |
|  | Typing speed | Two characters | 1^st^ spectral moment | -0.07 (-0.10, -0.04) | <0.001 |
|  | Typing speed | Two characters | 2^nd^ spectral moment | -0.08 (-0.11, -0.04) | <0.001 |
|  | Typing speed | Two characters | 3^rd^ spectral moment | -0.08 (-0.11, -0.05) | <0.001 |
|  | Typing speed | Two characters | Total (sum) power | -0.08 (-0.11, -0.05) | <0.001 |
|  | Typing speed | Two characters | Median frequency | -0.08 (-0.11, -0.05) | <0.001 |
|  | Typing speed | Two characters | Mean frequency | -0.08 (-0.12, -0.05) | <0.001 |
|  | Typing speed | Two characters | 1^st^ spectral moment | -0.08 (-0.11, -0.05) | <0.001 |
|  | Typing speed | Two characters | 2^nd^ spectral moment | -0.09 (-0.12, -0.05) | <0.001 |
|  | Typing speed | Two characters | 3^rd^ spectral moment | -0.09 (-0.12, -0.06) | <0.001 |
|  | Typing speed | Consecutive typing events | Mean log | 0.07 (0.04, 0.10) | <0.001 |
|  | Typing speed | Consecutive typing events | 50^th^ percentile | 0.06 (0.03, 0.10) | <0.001 |
|  | Typing speed | Consecutive typing events | 80^th^ percentile | 0.07 (0.04, 0.10) | <0.001 |
|  | Typing speed | Consecutive typing events | Total (sum) power | -0.08 (-0.11, -0.05) | <0.001 |
|  | Typing speed | Consecutive typing events | Median frequency | -0.07 (-0.10, -0.04) | <0.001 |
|  | Typing speed | Consecutive typing events | Mean frequency | -0.09 (-0.12, -0.05) | <0.001 |
|  | Typing speed | Consecutive typing events | 1^st^ spectral moment | -0.08 (-0.11, -0.05) | <0.001 |
|  | Typing speed | Consecutive typing events | 2^nd^ spectral moment | -0.08 (-0.12, -0.05) | <0.001 |
|  | Typing speed | Consecutive typing events | 3^rd^ spectral moment | -0.08 (-0.12, -0.05) | <0.001 |
|  | Typing speed | ≤ 3 characters in a row | Total (sum) power | -0.06 (-0.10, -0.03) | <0.001 |
|  | Typing speed | ≤ 3 characters in a row | Mean frequency | -0.07 (-0.10, -0.03) | <0.001 |
|  | Typing speed | ≤ 3 characters in a row | 1^st^ spectral moment | -0.07 (-0.10, -0.04) | <0.001 |
|  | Typing speed | ≤ 3 characters in a row | 2^nd^ spectral moment | -0.07 (-0.10, -0.04) | <0.001 |
|  | Typing speed | ≤ 3 characters in a row | 3^rd^ spectral moment | -0.07 (-0.10, -0.04) | <0.001 |
|  | Typing speed | > 3 characters in a row | Mean log | 0.07 (0.04, 0.11) | <0.001 |
|  | Typing speed | > 3 characters in a row | 10^th^ percentile | 0.06 (0.03, 0.10) | <0.001 |
|  | Typing speed | > 3 characters in a row | 20^th^ percentile | 0.06 (0.03, 0.09) | <0.001 |
|  | Typing speed | > 3 characters in a row | 50^th^ percentile | 0.07 (0.04, 0.10) | <0.001 |
|  | Typing speed | > 3 characters in a row | 80^th^ percentile | 0.07 (0.04, 0.10) | <0.001 |
|  | Typing speed | > 3 characters in a row | 90^th^ percentile | 0.06 (0.03, 0.09) | <0.001 |
|  | Typing speed | > 3 characters in a row | Total (sum) power | -0.09 (-0.12, -0.06) | <0.001 |
|  | Typing speed | > 3 characters in a row | Median frequency | -0.09 (-0.12, -0.06) | <0.001 |
|  | Typing speed | > 3 characters in a row | Mean frequency | -0.10 (-0.13, -0.07) | <0.001 |
|  | Typing speed | > 3 characters in a row | 1^st^ spectral moment | -0.09 (-0.12, -0.06) | <0.001 |
|  | Typing speed | > 3 characters in a row | 2^nd^ spectral moment | -0.10 (-0.13, -0.07) | <0.001 |
|  | Typing speed | > 3 characters in a row | 3^rd^ spectral moment | -0.10 (-0.13, -0.07) | <0.001 |
| Difficulty with concentration/thinking/fatigue | Scroll | Speed of scroll to item | 50^th^ percentile | 0.06 (0.03, 0.10) | <0.001 |
|  | Scroll | Speed of scroll to item | Median frequency | -0.07 (-0.10, -0.04) | <0.001 |
|  | Scroll | Speed of scroll to item | Mean frequency | -0.07 (-0.10, -0.03) | <0.001 |
|  | Scroll | Speed of scroll to item | 3^rd^ spectral moment | -0.06 (-0.09, -0.03) | <0.001 |
|  | Typing speed | > 3 characters in a row | Mean frequency | -0.07 (-0.10, -0.04) | <0.001 |
|  | Typing speed | > 3 characters in a row | 1^st^ spectral moment | -0.06 (-0.09, -0.03) | <0.001 |
|  | Typing speed | > 3 characters in a row | 2^nd^ spectral moment | -0.07 (-0.10, -0.03) | <0.001 |
|  | Typing speed | > 3 characters in a row | 3^rd^ spectral moment | -0.07 (-0.10, -0.03) | <0.001 |

**Supplementary Table 6.**

Prediction of worsening adverse posttraumatic neuropsychiatric symptoms using state keystroke biomarkers captured via mobile application among trauma survivors participating in an observational cohort study.

| **Construct** | **N (%) Worsening** | **Theme** | **Type** | **Signal Processing** | **Sensitivity** | **Specificity** | **PPV** | **NPV** | **Accuracy** |
| --- | --- | --- | --- | --- | --- | --- | --- | --- | --- |
| Pain | 159 (23%) | Typing speed | Two characters in a row | Total (sum) power | 0.41 | 0.61 | 0.24 | 0.78 | 0.57 |
|  |  | Typing speed | Two characters in a row | Mean frequency | 0.38 | 0.6 | 0.22 | 0.76 | 0.55 |
|  |  | Typing speed | Two characters in a row | 1^st^ spectral moment | 0.39 | 0.61 | 0.23 | 0.77 | 0.56 |
|  |  | Typing speed | Two characters in a row | 2^nd^ spectral moment | 0.41 | 0.61 | 0.24 | 0.77 | 0.56 |
|  |  | Typing speed | Two characters in a row | 3^rd^ spectral moment | 0.40 | 0.60 | 0.23 | 0.77 | 0.55 |
|  |  | Typing speed | Two characters | Total (sum) power | 0.39 | 0.59 | 0.22 | 0.77 | 0.55 |
|  |  | Typing speed | Two characters | Mean frequency | 0.36 | 0.65 | 0.23 | 0.77 | 0.58 |
|  |  | Typing speed | Two characters | 1^st^ spectral moment | 0.38 | 0.61 | 0.22 | 0.77 | 0.55 |
|  |  | Typing speed | Two characters | 2^nd^ spectral moment | 0.38 | 0.61 | 0.22 | 0.77 | 0.56 |
|  |  | Typing speed | Two characters | 3^rd^ spectral moment | 0.38 | 0.62 | 0.23 | 0.77 | 0.56 |
|  |  | Typing speed | Two characters | Total (sum) power | 0.43 | 0.62 | 0.25 | 0.79 | 0.57 |
|  |  | Typing speed | Two characters | Mean frequency | 0.36 | 0.62 | 0.22 | 0.76 | 0.56 |
|  |  | Typing speed | Two characters | 1^st^ spectral moment | 0.42 | 0.62 | 0.25 | 0.78 | 0.57 |
|  |  | Typing speed | Two characters | 2^nd^ spectral moment | 0.43 | 0.62 | 0.25 | 0.78 | 0.57 |
|  |  | Typing speed | Two characters | 3^rd^ spectral moment | 0.41 | 0.63 | 0.25 | 0.78 | 0.58 |
|  |  | Typing speed | Consecutive typing events | Mean log | 0.37 | 0.65 | 0.24 | 0.78 | 0.58 |
|  |  | Typing speed | Consecutive typing events | 20^th^ percentile | 0.42 | 0.63 | 0.25 | 0.78 | 0.58 |
|  |  | Typing speed | Consecutive typing events | 50^th^ percentile | 0.36 | 0.64 | 0.23 | 0.77 | 0.58 |
|  |  | Typing speed | Consecutive typing events | 80^th^ percentile | 0.39 | 0.60 | 0.23 | 0.77 | 0.55 |
|  |  | Typing speed | Consecutive typing events | 90^th^ percentile | 0.41 | 0.54 | 0.21 | 0.75 | 0.51 |
|  |  | Typing speed | Consecutive typing events | Total (sum) power | 0.39 | 0.61 | 0.23 | 0.77 | 0.56 |
|  |  | Typing speed | Consecutive typing events | Median frequency | 0.39 | 0.60 | 0.23 | 0.77 | 0.55 |
|  |  | Typing speed | Consecutive typing events | Mean frequency | 0.39 | 0.62 | 0.23 | 0.77 | 0.57 |
|  |  | Typing speed | Consecutive typing events | 1^st^ spectral moment | 0.41 | 0.62 | 0.24 | 0.78 | 0.57 |
|  |  | Typing speed | Consecutive typing events | 2^nd^ spectral moment | 0.42 | 0.61 | 0.25 | 0.78 | 0.57 |
|  |  | Typing speed | Consecutive typing events | 3^rd^ spectral moment | 0.44 | 0.61 | 0.25 | 0.79 | 0.57 |
|  |  | Scroll | Scroll twice to item | Maximum fractal length | 0.36 | 0.59 | 0.21 | 0.75 | 0.54 |
|  |  | Typing speed | ≤ 3 characters in a row | Mean frequency | 0.44 | 0.58 | 0.24 | 0.77 | 0.54 |
|  |  | Typing speed | > 3 characters in a row | Mean frequency | 0.42 | 0.55 | 0.22 | 0.77 | 0.52 |
|  |  | Typing speed | > 3 characters in a row | 1^st^ spectral moment | 0.46 | 0.58 | 0.24 | 0.78 | 0.55 |
|  |  | Typing speed | > 3 characters in a row | 2^nd^ spectral moment | 0.46 | 0.56 | 0.23 | 0.78 | 0.54 |
|  |  | Typing speed | > 3 characters in a row | 3^rd^ spectral moment | 0.46 | 0.54 | 0.23 | 0.77 | 0.52 |
|  |  | Composite biomarker | | | 0.29 | 0.55 | 0.16 | 0.73 | 0.49 |
| Depressive symptoms | 237 (47%) | Typing speed | The first set of at least 5 characters in a row | Maximum fractal length | 0.50 | 0.50 | 0.47 | 0.53 | 0.50 |
| Sleep Disturbance | 269 (55%) | Typing speed | Two characters in a row | Mean log | 0.50 | 0.48 | 0.54 | 0.44 | 0.49 |
|  |  | Typing speed | Two characters in a row | Total (sum) power | 0.51 | 0.47 | 0.54 | 0.44 | 0.49 |
|  |  | Typing speed | Two characters in a row | Mean frequency | 0.53 | 0.46 | 0.55 | 0.44 | 0.50 |
|  |  | Typing speed | Two characters in a row | 1^st^ spectral moment | 0.51 | 0.49 | 0.55 | 0.45 | 0.50 |
|  |  | Typing speed | Two characters in a row | 2^nd^ spectral moment | 0.52 | 0.48 | 0.55 | 0.45 | 0.50 |
|  |  | Typing speed | Two characters in a row | 3^rd^ spectral moment | 0.53 | 0.48 | 0.56 | 0.45 | 0.51 |
|  |  | Typing speed | Two characters | Mean log | 0.51 | 0.5 | 0.55 | 0.45 | 0.5 |
|  |  | Typing speed | Two characters | 20^th^ percentile | 0.52 | 0.47 | 0.55 | 0.44 | 0.5 |
|  |  | Typing speed | Two characters | Total (sum) power | 0.48 | 0.49 | 0.54 | 0.43 | 0.49 |
|  |  | Typing speed | Two characters | Mean frequency | 0.51 | 0.49 | 0.55 | 0.45 | 0.5 |
|  |  | Typing speed | Two characters | 1^st^ spectral moment | 0.49 | 0.49 | 0.54 | 0.44 | 0.49 |
|  |  | Typing speed | Two characters | 2^nd^ spectral moment | 0.5 | 0.48 | 0.54 | 0.44 | 0.49 |
|  |  | Typing speed | Two characters | 3^rd^ spectral moment | 0.51 | 0.48 | 0.55 | 0.44 | 0.5 |
|  |  | Typing speed | Two characters | Mean log | 0.5 | 0.5 | 0.55 | 0.45 | 0.5 |
|  |  | Typing speed | Two characters | 20^th^ percentile | 0.52 | 0.51 | 0.56 | 0.46 | 0.51 |
|  |  | Typing speed | Two characters | Total (sum) power | 0.52 | 0.49 | 0.55 | 0.45 | 0.51 |
|  |  | Typing speed | Two characters | Mean frequency | 0.55 | 0.49 | 0.57 | 0.47 | 0.53 |
|  |  | Typing speed | Two characters | 1^st^ spectral moment | 0.53 | 0.5 | 0.57 | 0.47 | 0.52 |
|  |  | Typing speed | Two characters | 2^nd^ spectral moment | 0.53 | 0.5 | 0.56 | 0.46 | 0.52 |
|  |  | Typing speed | Two characters | 3^rd^ spectral moment | 0.54 | 0.5 | 0.57 | 0.47 | 0.52 |
|  |  | Typing speed | Consecutive typing events | Mean | 0.5 | 0.5 | 0.55 | 0.45 | 0.5 |
|  |  | Typing speed | Consecutive typing events | Mean log | 0.52 | 0.52 | 0.57 | 0.47 | 0.52 |
|  |  | Typing speed | Consecutive typing events | 20^th^ percentile | 0.53 | 0.49 | 0.56 | 0.46 | 0.51 |
|  |  | Typing speed | Consecutive typing events | 50^th^ percentile | 0.57 | 0.5 | 0.59 | 0.49 | 0.54 |
|  |  | Typing speed | Consecutive typing events | 80^th^ percentile | 0.54 | 0.52 | 0.58 | 0.48 | 0.53 |
|  |  | Typing speed | Consecutive typing events | 90^th^ percentile | 0.51 | 0.49 | 0.55 | 0.44 | 0.5 |
|  |  | Typing speed | Consecutive typing events | Total (sum) power | 0.51 | 0.51 | 0.56 | 0.46 | 0.51 |
|  |  | Typing speed | Consecutive typing events | Mean frequency | 0.52 | 0.51 | 0.57 | 0.46 | 0.52 |
|  |  | Typing speed | Consecutive typing events | 1^st^ spectral moment | 0.50 | 0.51 | 0.56 | 0.46 | 0.51 |
|  |  | Typing speed | Consecutive typing events | 2^nd^ spectral moment | 0.51 | 0.51 | 0.56 | 0.46 | 0.51 |
|  |  | Typing speed | Consecutive typing events | 3^rd^ spectral moment | 0.51 | 0.50 | 0.56 | 0.46 | 0.51 |
|  |  | Typing speed | > 3 characters in a row | Mean | 0.52 | 0.48 | 0.56 | 0.44 | 0.5 |
|  |  | Typing speed | > 3 characters in a row | Mean log | 0.51 | 0.50 | 0.57 | 0.45 | 0.51 |
|  |  | Typing speed | > 3 characters in a row | 10^th^ percentile | 0.49 | 0.57 | 0.59 | 0.47 | 0.52 |
|  |  | Typing speed | > 3 characters in a row | 20^th^ percentile | 0.53 | 0.54 | 0.59 | 0.47 | 0.53 |
|  |  | Typing speed | > 3 characters in a row | 50^th^ percentile | 0.53 | 0.48 | 0.56 | 0.45 | 0.51 |
|  |  | Typing speed | > 3 characters in a row | 80^th^ percentile | 0.50 | 0.5 | 0.56 | 0.44 | 0.50 |
|  |  | Typing speed | > 3 characters in a row | 90^th^ percentile | 0.46 | 0.47 | 0.52 | 0.41 | 0.46 |
|  |  | Typing speed | > 3 characters in a row | Mean power | 0.50 | 0.46 | 0.54 | 0.42 | 0.49 |
|  |  | Typing speed | > 3 characters in a row | Total (sum) power | 0.52 | 0.47 | 0.55 | 0.43 | 0.50 |
|  |  | Typing speed | > 3 characters in a row | Mean frequency | 0.50 | 0.50 | 0.56 | 0.44 | 0.50 |
|  |  | Typing speed | > 3 characters in a row | 1^st^ spectral moment | 0.52 | 0.49 | 0.56 | 0.44 | 0.50 |
|  |  | Typing speed | > 3 characters in a row | 2^nd^ spectral moment | 0.52 | 0.48 | 0.56 | 0.44 | 0.50 |
|  |  | Typing speed | > 3 characters in a row | 3^rd^ spectral moment | 0.51 | 0.49 | 0.56 | 0.44 | 0.50 |
|  |  | Composite biomarker | | | 0.57 | 0.52 | 0.61 | 0.47 | 0.55 |
| Hyperarousal | 263 (52%) | Typing speed | Character to space | 1^st^ spectral moment | 0.52 | 0.47 | 0.53 | 0.47 | 0.50 |
|  |  | Typing speed | Character to space | 2^nd^ spectral moment | 0.52 | 0.48 | 0.53 | 0.47 | 0.50 |
|  |  | Typing speed | Character to space | 3^rd^ spectral moment | 0.51 | 0.47 | 0.52 | 0.46 | 0.49 |
|  |  | Typing speed | Two characters in a row | Total (sum) power | 0.50 | 0.50 | 0.52 | 0.48 | 0.50 |
|  |  | Typing speed | Two characters in a row | Mean frequency | 0.55 | 0.51 | 0.55 | 0.51 | 0.53 |
|  |  | Typing speed | Two characters in a row | 1^st^ spectral moment | 0.51 | 0.49 | 0.52 | 0.49 | 0.50 |
|  |  | Typing speed | Two characters in a row | 2^nd^ spectral moment | 0.53 | 0.49 | 0.52 | 0.49 | 0.51 |
|  |  | Typing speed | Two characters in a row | 3^rd^ spectral moment | 0.52 | 0.50 | 0.53 | 0.49 | 0.51 |
|  |  | Typing speed | Character to space | Total (sum) power | 0.53 | 0.52 | 0.55 | 0.5 | 0.53 |
|  |  | Typing speed | Character to space | Mean frequency | 0.54 | 0.51 | 0.55 | 0.5 | 0.53 |
|  |  | Typing speed | Character to space | 1^st^ spectral moment | 0.51 | 0.52 | 0.55 | 0.49 | 0.52 |
|  |  | Typing speed | Character to space | 2^nd^ spectral moment | 0.52 | 0.52 | 0.55 | 0.49 | 0.52 |
|  |  | Typing speed | Character to space | 3^rd^ spectral moment | 0.52 | 0.52 | 0.55 | 0.49 | 0.52 |
|  |  | Typing speed | Two characters | 1^st^ spectral moment | 0.50 | 0.50 | 0.52 | 0.48 | 0.50 |
|  |  | Typing speed | Two characters | 2^nd^ spectral moment | 0.50 | 0.51 | 0.53 | 0.49 | 0.51 |
|  |  | Typing speed | Two characters | 3^rd^ spectral moment | 0.53 | 0.51 | 0.53 | 0.50 | 0.52 |
|  |  | Typing speed | Consecutive typing events | 1^st^ spectral moment | 0.51 | 0.50 | 0.52 | 0.49 | 0.51 |
|  |  | Typing speed | Consecutive typing events | 2^nd^ spectral moment | 0.50 | 0.50 | 0.52 | 0.48 | 0.50 |
|  |  | Typing speed | Consecutive typing events | 3^rd^ spectral moment | 0.50 | 0.50 | 0.52 | 0.48 | 0.50 |
|  |  | Typing speed | > 3 characters in a row | Mean log | 0.55 | 0.49 | 0.54 | 0.50 | 0.52 |
|  |  | Typing speed | > 3 characters in a row | 20^th^ percentile | 0.51 | 0.47 | 0.51 | 0.47 | 0.49 |
|  |  | Typing speed | > 3 characters in a row | Total (sum) power | 0.54 | 0.47 | 0.53 | 0.48 | 0.51 |
|  |  | Typing speed | > 3 characters in a row | Mean frequency | 0.53 | 0.48 | 0.53 | 0.48 | 0.51 |
|  |  | Typing speed | > 3 characters in a row | 1^st^ spectral moment | 0.54 | 0.47 | 0.53 | 0.48 | 0.51 |
|  |  | Typing speed | > 3 characters in a row | 2^nd^ spectral moment | 0.55 | 0.47 | 0.53 | 0.48 | 0.51 |
|  |  | Typing speed | > 3 characters in a row | 3^rd^ spectral moment | 0.55 | 0.47 | 0.53 | 0.49 | 0.51 |
|  |  | Typing speed | The first set of at least 5 characters in a row | Standard deviation of the difference in differences | 0.57 | 0.49 | 0.60 | 0.46 | 0.53 |
|  |  | Typing speed | The first set of at least 5 characters in a row | Maximum fractal length | 0.54 | 0.55 | 0.62 | 0.48 | 0.55 |
|  |  | Typing speed | The first set of at least 5 characters in a row | Total (sum) power | 0.56 | 0.51 | 0.60 | 0.46 | 0.54 |
|  |  | Typing speed | The first set of at least 5 characters in a row | 1^st^ spectral moment | 0.57 | 0.50 | 0.60 | 0.47 | 0.54 |
|  |  | Typing speed | The first set of at least 5 characters in a row | 2^nd^ spectral moment | 0.57 | 0.52 | 0.61 | 0.47 | 0.55 |
|  |  | Typing speed | The first set of at least 5 characters in a row | 3^rd^ spectral moment | 0.57 | 0.51 | 0.61 | 0.47 | 0.54 |
|  |  | Typing speed | The second set of at least 5 characters in a row | Mean | 0.53 | 0.50 | 0.59 | 0.45 | 0.52 |
|  |  | Typing speed | The second set of at least 5 characters in a row | Mean log | 0.53 | 0.46 | 0.56 | 0.43 | 0.50 |
|  |  | Typing speed | The second set of at least 5 characters in a row | 10^th^ percentile | 0.49 | 0.51 | 0.57 | 0.43 | 0.50 |
|  |  | Typing speed | The second set of at least 5 characters in a row | 20^th^ percentile | 0.50 | 0.51 | 0.57 | 0.43 | 0.50 |
|  |  | Typing speed | The second set of at least 5 characters in a row | 50^th^ percentile | 0.51 | 0.50 | 0.57 | 0.44 | 0.51 |
|  |  | Typing speed | The second set of at least 5 characters in a row | 80^th^ percentile | 0.53 | 0.52 | 0.59 | 0.46 | 0.53 |
|  |  | Typing speed | The second set of at least 5 characters in a row | Mean power | 0.52 | 0.53 | 0.59 | 0.46 | 0.53 |
|  |  | Typing speed | The second set of at least 5 characters in a row | Total (sum) power | 0.51 | 0.48 | 0.57 | 0.43 | 0.50 |
|  |  | Typing speed | The second set of at least 5 characters in a row | Mean frequency | 0.53 | 0.49 | 0.58 | 0.44 | 0.51 |
|  |  | Typing speed | The second set of at least 5 characters in a row | 1^st^ spectral moment | 0.51 | 0.48 | 0.56 | 0.43 | 0.50 |
|  |  | Typing speed | The second set of at least 5 characters in a row | 2^nd^ spectral moment | 0.51 | 0.48 | 0.56 | 0.43 | 0.50 |
|  |  | Typing speed | The second set of at least 5 characters in a row | 3^rd^ spectral moment | 0.52 | 0.48 | 0.57 | 0.44 | 0.51 |
|  |  | Composite biomarker | | | 0.52 | 0.49 | 0.49 | 0.52 | 0.51 |
| Reexperiencing | 226 (47%) | Typing speed | Character to space | Mean log | 0.55 | 0.49 | 0.48 | 0.56 | 0.52 |
|  |  | Typing speed | Character to space | 10^th^ percentile | 0.58 | 0.51 | 0.50 | 0.59 | 0.54 |
|  |  | Typing speed | Character to space | 20^th^ percentile | 0.56 | 0.50 | 0.49 | 0.57 | 0.53 |
|  |  | Typing speed | Character to space | 90^th^ percentile | 0.53 | 0.48 | 0.46 | 0.55 | 0.50 |
|  |  | Typing speed | Character to space | Total (sum) power | 0.55 | 0.49 | 0.48 | 0.56 | 0.52 |
|  |  | Typing speed | Character to space | Mean frequency | 0.55 | 0.51 | 0.49 | 0.57 | 0.53 |
|  |  | Typing speed | Character to space | 1^st^ spectral moment | 0.54 | 0.48 | 0.47 | 0.55 | 0.51 |
|  |  | Typing speed | Character to space | 2^nd^ spectral moment | 0.55 | 0.49 | 0.48 | 0.56 | 0.52 |
|  |  | Typing speed | Character to space | 3^rd^ spectral moment | 0.55 | 0.49 | 0.48 | 0.56 | 0.52 |
|  |  | Typing speed | Character to space | 10^th^ percentile | 0.56 | 0.49 | 0.48 | 0.57 | 0.52 |
|  |  | Typing speed | Character to space | Total (sum) power | 0.57 | 0.50 | 0.49 | 0.58 | 0.53 |
|  |  | Typing speed | Character to space | Median frequency | 0.55 | 0.54 | 0.5 | 0.59 | 0.54 |
|  |  | Typing speed | Character to space | Mean frequency | 0.56 | 0.50 | 0.48 | 0.58 | 0.53 |
|  |  | Typing speed | Character to space | 1^st^ spectral moment | 0.56 | 0.49 | 0.48 | 0.57 | 0.52 |
|  |  | Typing speed | Character to space | 2^nd^ spectral moment | 0.56 | 0.50 | 0.49 | 0.58 | 0.53 |
|  |  | Typing speed | Character to space | 3^rd^ spectral moment | 0.57 | 0.50 | 0.49 | 0.58 | 0.53 |
|  |  | Typing speed | Two characters | Mean frequency | 0.53 | 0.50 | 0.49 | 0.54 | 0.51 |
|  |  | Typing speed | Consecutive typing events | Mean log | 0.51 | 0.46 | 0.46 | 0.52 | 0.49 |
|  |  | Typing speed | Consecutive typing events | Total (sum) power | 0.50 | 0.48 | 0.46 | 0.52 | 0.49 |
|  |  | Typing speed | Consecutive typing events | Mean frequency | 0.47 | 0.47 | 0.44 | 0.50 | 0.47 |
|  |  | Typing speed | Consecutive typing events | 1^st^ spectral moment | 0.50 | 0.48 | 0.46 | 0.52 | 0.49 |
|  |  | Typing speed | Consecutive typing events | 2^nd^ spectral moment | 0.49 | 0.50 | 0.47 | 0.52 | 0.50 |
|  |  | Typing speed | Consecutive typing events | 3^rd^ spectral moment | 0.50 | 0.50 | 0.47 | 0.53 | 0.50 |
|  |  | Typing speed | ≤ 3 characters in a row | Total (sum) power | 0.54 | 0.51 | 0.49 | 0.55 | 0.52 |
|  |  | Typing speed | ≤ 4 characters then space | Mean frequency | 0.56 | 0.47 | 0.48 | 0.56 | 0.52 |
|  |  | Typing speed | ≤ 4 characters then space | 1^st^ spectral moment | 0.56 | 0.48 | 0.48 | 0.56 | 0.52 |
|  |  | Typing speed | ≤ 4 characters then space | 2^nd^ spectral moment | 0.56 | 0.47 | 0.48 | 0.56 | 0.52 |
|  |  | Typing speed | ≤ 4 characters then space | 3^rd^ spectral moment | 0.58 | 0.48 | 0.49 | 0.57 | 0.53 |
|  |  | Composite biomarker | | | 0.54 | 0.48 | 0.51 | 0.51 | 0.51 |
| Somatic Symptoms | 130 (23%) | Typing speed | Two characters in a row | 80^th^ percentile | 0.46 | 0.55 | 0.23 | 0.78 | 0.53 |
|  |  | Typing speed | Two characters in a row | 90^th^ percentile | 0.45 | 0.51 | 0.22 | 0.76 | 0.50 |
|  |  | Typing speed | Two characters in a row | Total (sum) power | 0.46 | 0.54 | 0.23 | 0.77 | 0.52 |
|  |  | Typing speed | Two characters in a row | Median frequency | 0.44 | 0.55 | 0.22 | 0.77 | 0.53 |
|  |  | Typing speed | Two characters in a row | Mean frequency | 0.48 | 0.57 | 0.25 | 0.79 | 0.55 |
|  |  | Typing speed | Two characters in a row | 1^st^ spectral moment | 0.47 | 0.53 | 0.23 | 0.77 | 0.52 |
|  |  | Typing speed | Two characters in a row | 2^nd^ spectral moment | 0.45 | 0.53 | 0.22 | 0.77 | 0.51 |
|  |  | Typing speed | Two characters in a row | 3^rd^ spectral moment | 0.46 | 0.53 | 0.23 | 0.77 | 0.52 |
|  |  | Typing speed | Two characters | Total (sum) power | 0.48 | 0.52 | 0.23 | 0.78 | 0.52 |
|  |  | Typing speed | Two characters | Median frequency | 0.43 | 0.55 | 0.22 | 0.77 | 0.52 |
|  |  | Typing speed | Two characters | Mean frequency | 0.42 | 0.54 | 0.21 | 0.76 | 0.51 |
|  |  | Typing speed | Two characters | 1^st^ spectral moment | 0.48 | 0.53 | 0.23 | 0.78 | 0.52 |
|  |  | Typing speed | Two characters | 2^nd^ spectral moment | 0.47 | 0.53 | 0.23 | 0.77 | 0.52 |
|  |  | Typing speed | Two characters | 3^rd^ spectral moment | 0.48 | 0.53 | 0.23 | 0.78 | 0.52 |
|  |  | Typing speed | Two characters | Total (sum) power | 0.48 | 0.54 | 0.23 | 0.78 | 0.52 |
|  |  | Typing speed | Two characters | Median frequency | 0.45 | 0.54 | 0.22 | 0.77 | 0.52 |
|  |  | Typing speed | Two characters | Mean frequency | 0.45 | 0.54 | 0.22 | 0.77 | 0.52 |
|  |  | Typing speed | Two characters | 1^st^ spectral moment | 0.51 | 0.53 | 0.24 | 0.79 | 0.53 |
|  |  | Typing speed | Two characters | 2^nd^ spectral moment | 0.51 | 0.53 | 0.24 | 0.79 | 0.53 |
|  |  | Typing speed | Two characters | 3^rd^ spectral moment | 0.49 | 0.54 | 0.24 | 0.78 | 0.53 |
|  |  | Typing speed | Consecutive typing events | Mean log | 0.48 | 0.56 | 0.24 | 0.78 | 0.54 |
|  |  | Typing speed | Consecutive typing events | 50^th^ percentile | 0.45 | 0.55 | 0.23 | 0.77 | 0.52 |
|  |  | Typing speed | Consecutive typing events | 80^th^ percentile | 0.47 | 0.54 | 0.23 | 0.78 | 0.53 |
|  |  | Typing speed | Consecutive typing events | Total (sum) power | 0.48 | 0.56 | 0.25 | 0.79 | 0.54 |
|  |  | Typing speed | Consecutive typing events | Median frequency | 0.40 | 0.56 | 0.21 | 0.76 | 0.52 |
|  |  | Typing speed | Consecutive typing events | Mean frequency | 0.43 | 0.54 | 0.22 | 0.76 | 0.51 |
|  |  | Typing speed | Consecutive typing events | 1^st^ spectral moment | 0.48 | 0.57 | 0.25 | 0.79 | 0.55 |
|  |  | Typing speed | Consecutive typing events | 2^nd^ spectral moment | 0.47 | 0.57 | 0.24 | 0.79 | 0.55 |
|  |  | Typing speed | Consecutive typing events | 3^rd^ spectral moment | 0.46 | 0.57 | 0.24 | 0.78 | 0.54 |
|  |  | Typing speed | ≤ 3 characters in a row | Total (sum) power | 0.49 | 0.56 | 0.25 | 0.79 | 0.54 |
|  |  | Typing speed | ≤ 3 characters in a row | Mean frequency | 0.49 | 0.54 | 0.24 | 0.78 | 0.53 |
|  |  | Typing speed | ≤ 3 characters in a row | 1^st^ spectral moment | 0.50 | 0.56 | 0.25 | 0.79 | 0.54 |
|  |  | Typing speed | ≤ 3 characters in a row | 2^nd^ spectral moment | 0.50 | 0.55 | 0.25 | 0.79 | 0.54 |
|  |  | Typing speed | ≤ 3 characters in a row | 3^rd^ spectral moment | 0.50 | 0.55 | 0.25 | 0.79 | 0.54 |
|  |  | Typing speed | > 3 characters in a row | Mean log | 0.54 | 0.55 | 0.26 | 0.80 | 0.55 |
|  |  | Typing speed | > 3 characters in a row | 10^th^ percentile | 0.54 | 0.58 | 0.28 | 0.81 | 0.57 |
|  |  | Typing speed | > 3 characters in a row | 20^th^ percentile | 0.50 | 0.57 | 0.26 | 0.80 | 0.56 |
|  |  | Typing speed | > 3 characters in a row | 50^th^ percentile | 0.54 | 0.53 | 0.25 | 0.80 | 0.53 |
|  |  | Typing speed | > 3 characters in a row | 80^th^ percentile | 0.50 | 0.54 | 0.24 | 0.78 | 0.53 |
|  |  | Typing speed | > 3 characters in a row | 90^th^ percentile | 0.47 | 0.54 | 0.23 | 0.78 | 0.53 |
|  |  | Typing speed | > 3 characters in a row | Total (sum) power | 0.53 | 0.54 | 0.26 | 0.80 | 0.54 |
|  |  | Typing speed | > 3 characters in a row | Median frequency | 0.47 | 0.54 | 0.24 | 0.78 | 0.53 |
|  |  | Typing speed | > 3 characters in a row | Mean frequency | 0.52 | 0.57 | 0.26 | 0.80 | 0.55 |
|  |  | Typing speed | > 3 characters in a row | 1^st^ spectral moment | 0.54 | 0.54 | 0.26 | 0.80 | 0.54 |
|  |  | Typing speed | > 3 characters in a row | 2^nd^ spectral moment | 0.54 | 0.55 | 0.26 | 0.80 | 0.55 |
|  |  | Typing speed | > 3 characters in a row | 3^rd^ spectral moment | 0.57 | 0.55 | 0.27 | 0.81 | 0.56 |
|  |  | Composite biomarker | | | 0.56 | 0.52 | 0.26 | 0.8 | 0.53 |
| Difficulty with concentration/thinking/fatigue | 160 (28%) | Scroll | Speed of scroll to item | 50^th^ percentile | 0.54 | 0.50 | 0.30 | 0.74 | 0.51 |
|  |  | Scroll | Speed of scroll to item | Median frequency | 0.56 | 0.50 | 0.30 | 0.75 | 0.52 |
|  |  | Scroll | Speed of scroll to item | Mean frequency | 0.53 | 0.51 | 0.29 | 0.74 | 0.51 |
|  |  | Scroll | Speed of scroll to item | 3^rd^ spectral moment | 0.52 | 0.50 | 0.28 | 0.73 | 0.51 |
|  |  | Typing speed | > 3 characters in a row | Mean frequency | 0.49 | 0.56 | 0.30 | 0.74 | 0.54 |
|  |  | Typing speed | > 3 characters in a row | 1^st^ spectral moment | 0.51 | 0.54 | 0.30 | 0.74 | 0.53 |
|  |  | Typing speed | > 3 characters in a row | 2^nd^ spectral moment | 0.53 | 0.55 | 0.31 | 0.75 | 0.54 |
|  |  | Typing speed | > 3 characters in a row | 3^rd^ spectral moment | 0.54 | 0.55 | 0.32 | 0.76 | 0.55 |
|  |  | Composite biomarker | | | 0.61 | 0.48 | 0.34 | 0.73 | 0.52 |

**Supplementary Table 7.**

Prediction of recovering adverse posttraumatic neuropsychiatric symptoms using state keystroke biomarkers captured via mobile application among trauma survivors participating in an observational cohort study.

| **Construct** | **N (%) Recovering** | **Theme** | **Type** | **Signal Processing** | **Sensitivity** | **Specificity** | **PPV** | **NPV** | **Accuracy** |
| --- | --- | --- | --- | --- | --- | --- | --- | --- | --- |
| Pain | 533 (77%) | Typing speed | Two characters in a row | Total (sum) power | 0.61 | 0.41 | 0.78 | 0.24 | 0.57 |
|  |  | Typing speed | Two characters in a row | Mean frequency | 0.60 | 0.38 | 0.76 | 0.22 | 0.55 |
|  |  | Typing speed | Two characters in a row | 1^st^ spectral moment | 0.61 | 0.39 | 0.77 | 0.23 | 0.56 |
|  |  | Typing speed | Two characters in a row | 2^nd^ spectral moment | 0.61 | 0.41 | 0.77 | 0.24 | 0.56 |
|  |  | Typing speed | Two characters in a row | 3^rd^ spectral moment | 0.60 | 0.40 | 0.77 | 0.23 | 0.55 |
|  |  | Typing speed | Two characters | Total (sum) power | 0.59 | 0.39 | 0.77 | 0.22 | 0.55 |
|  |  | Typing speed | Two characters | Mean frequency | 0.65 | 0.36 | 0.77 | 0.23 | 0.58 |
|  |  | Typing speed | Two characters | 1^st^ spectral moment | 0.61 | 0.38 | 0.77 | 0.22 | 0.55 |
|  |  | Typing speed | Two characters | 2^nd^ spectral moment | 0.61 | 0.38 | 0.77 | 0.22 | 0.56 |
|  |  | Typing speed | Two characters | 3^rd^ spectral moment | 0.62 | 0.38 | 0.77 | 0.23 | 0.56 |
|  |  | Typing speed | Two characters | Total (sum) power | 0.62 | 0.43 | 0.79 | 0.25 | 0.57 |
|  |  | Typing speed | Two characters | Mean frequency | 0.62 | 0.36 | 0.76 | 0.22 | 0.56 |
|  |  | Typing speed | Two characters | 1^st^ spectral moment | 0.62 | 0.42 | 0.78 | 0.25 | 0.57 |
|  |  | Typing speed | Two characters | 2^nd^ spectral moment | 0.62 | 0.43 | 0.78 | 0.25 | 0.57 |
|  |  | Typing speed | Two characters | 3^rd^ spectral moment | 0.63 | 0.41 | 0.78 | 0.25 | 0.58 |
|  |  | Typing speed | Consecutive typing events | Mean log | 0.65 | 0.37 | 0.78 | 0.24 | 0.58 |
|  |  | Typing speed | Consecutive typing events | 20^th^ percentile | 0.63 | 0.42 | 0.78 | 0.25 | 0.58 |
|  |  | Typing speed | Consecutive typing events | 50^th^ percentile | 0.64 | 0.36 | 0.77 | 0.23 | 0.58 |
|  |  | Typing speed | Consecutive typing events | 80^th^ percentile | 0.60 | 0.39 | 0.77 | 0.23 | 0.55 |
|  |  | Typing speed | Consecutive typing events | 90^th^ percentile | 0.54 | 0.41 | 0.75 | 0.21 | 0.51 |
|  |  | Typing speed | Consecutive typing events | Total (sum) power | 0.61 | 0.39 | 0.77 | 0.23 | 0.56 |
|  |  | Typing speed | Consecutive typing events | Median frequency | 0.60 | 0.39 | 0.77 | 0.23 | 0.55 |
|  |  | Typing speed | Consecutive typing events | Mean frequency | 0.62 | 0.39 | 0.77 | 0.23 | 0.57 |
|  |  | Typing speed | Consecutive typing events | 1^st^ spectral moment | 0.62 | 0.41 | 0.78 | 0.24 | 0.57 |
|  |  | Typing speed | Consecutive typing events | 2^nd^ spectral moment | 0.61 | 0.42 | 0.78 | 0.25 | 0.57 |
|  |  | Typing speed | Consecutive typing events | 3^rd^ spectral moment | 0.61 | 0.44 | 0.79 | 0.25 | 0.57 |
|  |  | Scroll | Scroll twice to item | Maximum fractal length | 0.59 | 0.36 | 0.75 | 0.21 | 0.54 |
|  |  | Typing speed | ≤ 3 characters in a row | Mean frequency | 0.58 | 0.44 | 0.77 | 0.24 | 0.54 |
|  |  | Typing speed | > 3 characters in a row | Mean frequency | 0.55 | 0.42 | 0.77 | 0.22 | 0.52 |
|  |  | Typing speed | > 3 characters in a row | 1^st^ spectral moment | 0.58 | 0.46 | 0.78 | 0.24 | 0.55 |
|  |  | Typing speed | > 3 characters in a row | 2^nd^ spectral moment | 0.56 | 0.46 | 0.78 | 0.23 | 0.54 |
|  |  | Typing speed | > 3 characters in a row | 3^rd^ spectral moment | 0.54 | 0.46 | 0.77 | 0.23 | 0.52 |
|  |  | Composite biomarker | | | 0.55 | 0.29 | 0.73 | 0.16 | 0.49 |
| Depressive symptoms | 270 (53%) | Typing speed | The first set of at least 5 characters in a row | Maximum fractal length | 0.50 | 0.50 | 0.53 | 0.47 | 0.50 |
| Sleep Disturbance | 218 (45%) | Typing speed | Two characters in a row | Mean log | 0.48 | 0.50 | 0.44 | 0.54 | 0.49 |
|  |  | Typing speed | Two characters in a row | Total (sum) power | 0.47 | 0.51 | 0.44 | 0.54 | 0.49 |
|  |  | Typing speed | Two characters in a row | Mean frequency | 0.46 | 0.53 | 0.44 | 0.55 | 0.50 |
|  |  | Typing speed | Two characters in a row | 1^st^ spectral moment | 0.49 | 0.51 | 0.45 | 0.55 | 0.50 |
|  |  | Typing speed | Two characters in a row | 2^nd^ spectral moment | 0.48 | 0.52 | 0.45 | 0.55 | 0.50 |
|  |  | Typing speed | Two characters in a row | 3^rd^ spectral moment | 0.48 | 0.53 | 0.45 | 0.56 | 0.51 |
|  |  | Typing speed | Two characters | Mean log | 0.50 | 0.51 | 0.45 | 0.55 | 0.50 |
|  |  | Typing speed | Two characters | 20^th^ percentile | 0.47 | 0.52 | 0.44 | 0.55 | 0.50 |
|  |  | Typing speed | Two characters | Total (sum) power | 0.49 | 0.48 | 0.43 | 0.54 | 0.49 |
|  |  | Typing speed | Two characters | Mean frequency | 0.49 | 0.51 | 0.45 | 0.55 | 0.5 |
|  |  | Typing speed | Two characters | 1^st^ spectral moment | 0.49 | 0.49 | 0.44 | 0.54 | 0.49 |
|  |  | Typing speed | Two characters | 2^nd^ spectral moment | 0.48 | 0.50 | 0.44 | 0.54 | 0.49 |
|  |  | Typing speed | Two characters | 3^rd^ spectral moment | 0.48 | 0.51 | 0.44 | 0.55 | 0.50 |
|  |  | Typing speed | Two characters | Mean log | 0.50 | 0.50 | 0.45 | 0.55 | 0.50 |
|  |  | Typing speed | Two characters | 20^th^ percentile | 0.51 | 0.52 | 0.46 | 0.56 | 0.51 |
|  |  | Typing speed | Two characters | Total (sum) power | 0.49 | 0.52 | 0.45 | 0.55 | 0.51 |
|  |  | Typing speed | Two characters | Mean frequency | 0.49 | 0.55 | 0.47 | 0.57 | 0.53 |
|  |  | Typing speed | Two characters | 1^st^ spectral moment | 0.50 | 0.53 | 0.47 | 0.57 | 0.52 |
|  |  | Typing speed | Two characters | 2^nd^ spectral moment | 0.50 | 0.53 | 0.46 | 0.56 | 0.52 |
|  |  | Typing speed | Two characters | 3^rd^ spectral moment | 0.50 | 0.54 | 0.47 | 0.57 | 0.52 |
|  |  | Typing speed | Consecutive typing events | Mean | 0.50 | 0.50 | 0.45 | 0.55 | 0.50 |
|  |  | Typing speed | Consecutive typing events | Mean log | 0.52 | 0.52 | 0.47 | 0.57 | 0.52 |
|  |  | Typing speed | Consecutive typing events | 20^th^ percentile | 0.49 | 0.53 | 0.46 | 0.56 | 0.51 |
|  |  | Typing speed | Consecutive typing events | 50^th^ percentile | 0.50 | 0.57 | 0.49 | 0.59 | 0.54 |
|  |  | Typing speed | Consecutive typing events | 80^th^ percentile | 0.52 | 0.54 | 0.48 | 0.58 | 0.53 |
|  |  | Typing speed | Consecutive typing events | 90^th^ percentile | 0.49 | 0.51 | 0.44 | 0.55 | 0.50 |
|  |  | Typing speed | Consecutive typing events | Total (sum) power | 0.51 | 0.51 | 0.46 | 0.56 | 0.51 |
|  |  | Typing speed | Consecutive typing events | Mean frequency | 0.51 | 0.52 | 0.46 | 0.57 | 0.52 |
|  |  | Typing speed | Consecutive typing events | 1^st^ spectral moment | 0.51 | 0.50 | 0.46 | 0.56 | 0.51 |
|  |  | Typing speed | Consecutive typing events | 2^nd^ spectral moment | 0.51 | 0.51 | 0.46 | 0.56 | 0.51 |
|  |  | Typing speed | Consecutive typing events | 3^rd^ spectral moment | 0.50 | 0.51 | 0.46 | 0.56 | 0.51 |
|  |  | Typing speed | > 3 characters in a row | Mean | 0.48 | 0.52 | 0.44 | 0.56 | 0.50 |
|  |  | Typing speed | > 3 characters in a row | Mean log | 0.5 | 0.51 | 0.45 | 0.57 | 0.51 |
|  |  | Typing speed | > 3 characters in a row | 10^th^ percentile | 0.57 | 0.49 | 0.47 | 0.59 | 0.52 |
|  |  | Typing speed | > 3 characters in a row | 20^th^ percentile | 0.54 | 0.53 | 0.47 | 0.59 | 0.53 |
|  |  | Typing speed | > 3 characters in a row | 50^th^ percentile | 0.48 | 0.53 | 0.45 | 0.56 | 0.51 |
|  |  | Typing speed | > 3 characters in a row | 80^th^ percentile | 0.5 | 0.5 | 0.44 | 0.56 | 0.50 |
|  |  | Typing speed | > 3 characters in a row | 90^th^ percentile | 0.47 | 0.46 | 0.41 | 0.52 | 0.46 |
|  |  | Typing speed | > 3 characters in a row | Mean power | 0.46 | 0.50 | 0.42 | 0.54 | 0.49 |
|  |  | Typing speed | > 3 characters in a row | Total (sum) power | 0.47 | 0.52 | 0.43 | 0.55 | 0.50 |
|  |  | Typing speed | > 3 characters in a row | Mean frequency | 0.50 | 0.50 | 0.44 | 0.56 | 0.50 |
|  |  | Typing speed | > 3 characters in a row | 1^st^ spectral moment | 0.49 | 0.52 | 0.44 | 0.56 | 0.50 |
|  |  | Typing speed | > 3 characters in a row | 2^nd^ spectral moment | 0.48 | 0.52 | 0.44 | 0.56 | 0.50 |
|  |  | Typing speed | > 3 characters in a row | 3^rd^ spectral moment | 0.49 | 0.51 | 0.44 | 0.56 | 0.50 |
|  |  | Composite biomarker | | | 0.52 | 0.57 | 0.47 | 0.61 | 0.55 |
| Hyperarousal | 246 (48%) | Typing speed | Character to space | 1^st^ spectral moment | 0.47 | 0.52 | 0.47 | 0.53 | 0.50 |
|  |  | Typing speed | Character to space | 2^nd^ spectral moment | 0.48 | 0.52 | 0.47 | 0.53 | 0.50 |
|  |  | Typing speed | Character to space | 3^rd^ spectral moment | 0.47 | 0.51 | 0.46 | 0.52 | 0.49 |
|  |  | Typing speed | Two characters in a row | Total (sum) power | 0.50 | 0.50 | 0.48 | 0.52 | 0.50 |
|  |  | Typing speed | Two characters in a row | Mean frequency | 0.51 | 0.55 | 0.51 | 0.55 | 0.53 |
|  |  | Typing speed | Two characters in a row | 1^st^ spectral moment | 0.49 | 0.51 | 0.49 | 0.52 | 0.50 |
|  |  | Typing speed | Two characters in a row | 2^nd^ spectral moment | 0.49 | 0.53 | 0.49 | 0.52 | 0.51 |
|  |  | Typing speed | Two characters in a row | 3^rd^ spectral moment | 0.50 | 0.52 | 0.49 | 0.53 | 0.51 |
|  |  | Typing speed | Character to space | Total (sum) power | 0.52 | 0.53 | 0.50 | 0.55 | 0.53 |
|  |  | Typing speed | Character to space | Mean frequency | 0.51 | 0.54 | 0.50 | 0.55 | 0.53 |
|  |  | Typing speed | Character to space | 1^st^ spectral moment | 0.52 | 0.51 | 0.49 | 0.55 | 0.52 |
|  |  | Typing speed | Character to space | 2^nd^ spectral moment | 0.52 | 0.52 | 0.49 | 0.55 | 0.52 |
|  |  | Typing speed | Character to space | 3^rd^ spectral moment | 0.52 | 0.52 | 0.49 | 0.55 | 0.52 |
|  |  | Typing speed | Two characters | 1^st^ spectral moment | 0.50 | 0.50 | 0.48 | 0.52 | 0.50 |
|  |  | Typing speed | Two characters | 2^nd^ spectral moment | 0.51 | 0.50 | 0.49 | 0.53 | 0.51 |
|  |  | Typing speed | Two characters | 3^rd^ spectral moment | 0.51 | 0.53 | 0.5 | 0.53 | 0.52 |
|  |  | Typing speed | Consecutive typing events | 1^st^ spectral moment | 0.50 | 0.51 | 0.49 | 0.52 | 0.51 |
|  |  | Typing speed | Consecutive typing events | 2^nd^ spectral moment | 0.50 | 0.50 | 0.48 | 0.52 | 0.50 |
|  |  | Typing speed | Consecutive typing events | 3^rd^ spectral moment | 0.50 | 0.50 | 0.48 | 0.52 | 0.50 |
|  |  | Typing speed | > 3 characters in a row | Mean log | 0.49 | 0.55 | 0.5 | 0.54 | 0.52 |
|  |  | Typing speed | > 3 characters in a row | 20^th^ percentile | 0.47 | 0.51 | 0.47 | 0.51 | 0.49 |
|  |  | Typing speed | > 3 characters in a row | Total (sum) power | 0.47 | 0.54 | 0.48 | 0.53 | 0.51 |
|  |  | Typing speed | > 3 characters in a row | Mean frequency | 0.48 | 0.53 | 0.48 | 0.53 | 0.51 |
|  |  | Typing speed | > 3 characters in a row | 1^st^ spectral moment | 0.47 | 0.54 | 0.48 | 0.53 | 0.51 |
|  |  | Typing speed | > 3 characters in a row | 2^nd^ spectral moment | 0.47 | 0.55 | 0.48 | 0.53 | 0.51 |
|  |  | Typing speed | > 3 characters in a row | 3^rd^ spectral moment | 0.47 | 0.55 | 0.49 | 0.53 | 0.51 |
|  |  | Typing speed | The first set of at least 5 characters in a row | Standard deviation of the difference in differences | 0.49 | 0.57 | 0.46 | 0.60 | 0.53 |
|  |  | Typing speed | The first set of at least 5 characters in a row | Maximum fractal length | 0.55 | 0.54 | 0.48 | 0.62 | 0.55 |
|  |  | Typing speed | The first set of at least 5 characters in a row | Total (sum) power | 0.51 | 0.56 | 0.46 | 0.60 | 0.54 |
|  |  | Typing speed | The first set of at least 5 characters in a row | 1^st^ spectral moment | 0.50 | 0.57 | 0.47 | 0.60 | 0.54 |
|  |  | Typing speed | The first set of at least 5 characters in a row | 2^nd^ spectral moment | 0.52 | 0.57 | 0.47 | 0.61 | 0.55 |
|  |  | Typing speed | The first set of at least 5 characters in a row | 3^rd^ spectral moment | 0.51 | 0.57 | 0.47 | 0.6 | 0.54 |
|  |  | Typing speed | The second set of at least 5 characters in a row | Mean | 0.50 | 0.53 | 0.45 | 0.59 | 0.52 |
|  |  | Typing speed | The second set of at least 5 characters in a row | Mean log | 0.46 | 0.53 | 0.43 | 0.56 | 0.50 |
|  |  | Typing speed | The second set of at least 5 characters in a row | 10^th^ percentile | 0.51 | 0.49 | 0.43 | 0.57 | 0.50 |
|  |  | Typing speed | The second set of at least 5 characters in a row | 20^th^ percentile | 0.51 | 0.50 | 0.43 | 0.57 | 0.50 |
|  |  | Typing speed | The second set of at least 5 characters in a row | 50^th^ percentile | 0.50 | 0.51 | 0.44 | 0.57 | 0.51 |
|  |  | Typing speed | The second set of at least 5 characters in a row | 80^th^ percentile | 0.52 | 0.53 | 0.46 | 0.59 | 0.53 |
|  |  | Typing speed | The second set of at least 5 characters in a row | Mean power | 0.53 | 0.52 | 0.46 | 0.59 | 0.53 |
|  |  | Typing speed | The second set of at least 5 characters in a row | Total (sum) power | 0.48 | 0.51 | 0.43 | 0.57 | 0.50 |
|  |  | Typing speed | The second set of at least 5 characters in a row | Mean frequency | 0.49 | 0.53 | 0.44 | 0.58 | 0.51 |
|  |  | Typing speed | The second set of at least 5 characters in a row | 1^st^ spectral moment | 0.48 | 0.51 | 0.43 | 0.56 | 0.50 |
|  |  | Typing speed | The second set of at least 5 characters in a row | 2^nd^ spectral moment | 0.48 | 0.51 | 0.43 | 0.56 | 0.50 |
|  |  | Typing speed | The second set of at least 5 characters in a row | 3^rd^ spectral moment | 0.48 | 0.52 | 0.44 | 0.57 | 0.51 |
|  |  | Composite biomarker | | | 0.49 | 0.52 | 0.52 | 0.49 | 0.51 |
| Reexperiencing | 253 (53%) | Typing speed | Character to space | Mean log | 0.49 | 0.55 | 0.56 | 0.48 | 0.52 |
|  |  | Typing speed | Character to space | 10^th^ percentile | 0.51 | 0.58 | 0.59 | 0.50 | 0.54 |
|  |  | Typing speed | Character to space | 20^th^ percentile | 0.50 | 0.56 | 0.57 | 0.49 | 0.53 |
|  |  | Typing speed | Character to space | 90^th^ percentile | 0.48 | 0.53 | 0.55 | 0.46 | 0.50 |
|  |  | Typing speed | Character to space | Total (sum) power | 0.49 | 0.55 | 0.56 | 0.48 | 0.52 |
|  |  | Typing speed | Character to space | Mean frequency | 0.51 | 0.55 | 0.57 | 0.49 | 0.53 |
|  |  | Typing speed | Character to space | 1^st^ spectral moment | 0.48 | 0.54 | 0.55 | 0.47 | 0.51 |
|  |  | Typing speed | Character to space | 2^nd^ spectral moment | 0.49 | 0.55 | 0.56 | 0.48 | 0.52 |
|  |  | Typing speed | Character to space | 3^rd^ spectral moment | 0.49 | 0.55 | 0.56 | 0.48 | 0.52 |
|  |  | Typing speed | Character to space | 10^th^ percentile | 0.49 | 0.56 | 0.57 | 0.48 | 0.52 |
|  |  | Typing speed | Character to space | Total (sum) power | 0.50 | 0.57 | 0.58 | 0.49 | 0.53 |
|  |  | Typing speed | Character to space | Median frequency | 0.54 | 0.55 | 0.59 | 0.50 | 0.54 |
|  |  | Typing speed | Character to space | Mean frequency | 0.50 | 0.56 | 0.58 | 0.48 | 0.53 |
|  |  | Typing speed | Character to space | 1^st^ spectral moment | 0.49 | 0.56 | 0.57 | 0.48 | 0.52 |
|  |  | Typing speed | Character to space | 2^nd^ spectral moment | 0.50 | 0.56 | 0.58 | 0.49 | 0.53 |
|  |  | Typing speed | Character to space | 3^rd^ spectral moment | 0.50 | 0.57 | 0.58 | 0.49 | 0.53 |
|  |  | Typing speed | Two characters | Mean frequency | 0.50 | 0.53 | 0.54 | 0.49 | 0.51 |
|  |  | Typing speed | Consecutive typing events | Mean log | 0.46 | 0.51 | 0.52 | 0.46 | 0.49 |
|  |  | Typing speed | Consecutive typing events | Total (sum) power | 0.48 | 0.50 | 0.52 | 0.46 | 0.49 |
|  |  | Typing speed | Consecutive typing events | Mean frequency | 0.47 | 0.47 | 0.50 | 0.44 | 0.47 |
|  |  | Typing speed | Consecutive typing events | 1^st^ spectral moment | 0.48 | 0.50 | 0.52 | 0.46 | 0.49 |
|  |  | Typing speed | Consecutive typing events | 2^nd^ spectral moment | 0.50 | 0.49 | 0.52 | 0.47 | 0.50 |
|  |  | Typing speed | Consecutive typing events | 3^rd^ spectral moment | 0.50 | 0.50 | 0.53 | 0.47 | 0.50 |
|  |  | Typing speed | ≤ 3 characters in a row | Total (sum) power | 0.51 | 0.54 | 0.55 | 0.49 | 0.52 |
|  |  | Typing speed | ≤ 4 characters then space | Mean frequency | 0.47 | 0.56 | 0.56 | 0.48 | 0.52 |
|  |  | Typing speed | ≤ 4 characters then space | 1^st^ spectral moment | 0.48 | 0.56 | 0.56 | 0.48 | 0.52 |
|  |  | Typing speed | ≤ 4 characters then space | 2^nd^ spectral moment | 0.47 | 0.56 | 0.56 | 0.48 | 0.52 |
|  |  | Typing speed | ≤ 4 characters then space | 3^rd^ spectral moment | 0.48 | 0.58 | 0.57 | 0.49 | 0.53 |
|  |  | Composite biomarker | | | 0.48 | 0.54 | 0.51 | 0.51 | 0.51 |
| Somatic Symptoms | 442 (77%) | Typing speed | Two characters in a row | 80^th^ percentile | 0.55 | 0.46 | 0.78 | 0.23 | 0.53 |
|  |  | Typing speed | Two characters in a row | 90^th^ percentile | 0.51 | 0.45 | 0.76 | 0.22 | 0.50 |
|  |  | Typing speed | Two characters in a row | Total (sum) power | 0.54 | 0.46 | 0.77 | 0.23 | 0.52 |
|  |  | Typing speed | Two characters in a row | Median frequency | 0.55 | 0.44 | 0.77 | 0.22 | 0.53 |
|  |  | Typing speed | Two characters in a row | Mean frequency | 0.57 | 0.48 | 0.79 | 0.25 | 0.55 |
|  |  | Typing speed | Two characters in a row | 1^st^ spectral moment | 0.53 | 0.47 | 0.77 | 0.23 | 0.52 |
|  |  | Typing speed | Two characters in a row | 2^nd^ spectral moment | 0.53 | 0.45 | 0.77 | 0.22 | 0.51 |
|  |  | Typing speed | Two characters in a row | 3^rd^ spectral moment | 0.53 | 0.46 | 0.77 | 0.23 | 0.52 |
|  |  | Typing speed | Two characters | Total (sum) power | 0.52 | 0.48 | 0.78 | 0.23 | 0.52 |
|  |  | Typing speed | Two characters | Median frequency | 0.55 | 0.43 | 0.77 | 0.22 | 0.52 |
|  |  | Typing speed | Two characters | Mean frequency | 0.54 | 0.42 | 0.76 | 0.21 | 0.51 |
|  |  | Typing speed | Two characters | 1^st^ spectral moment | 0.53 | 0.48 | 0.78 | 0.23 | 0.52 |
|  |  | Typing speed | Two characters | 2^nd^ spectral moment | 0.53 | 0.47 | 0.77 | 0.23 | 0.52 |
|  |  | Typing speed | Two characters | 3^rd^ spectral moment | 0.53 | 0.48 | 0.78 | 0.23 | 0.52 |
|  |  | Typing speed | Two characters | Total (sum) power | 0.54 | 0.48 | 0.78 | 0.23 | 0.52 |
|  |  | Typing speed | Two characters | Median frequency | 0.54 | 0.45 | 0.77 | 0.22 | 0.52 |
|  |  | Typing speed | Two characters | Mean frequency | 0.54 | 0.45 | 0.77 | 0.22 | 0.52 |
|  |  | Typing speed | Two characters | 1^st^ spectral moment | 0.53 | 0.51 | 0.79 | 0.24 | 0.53 |
|  |  | Typing speed | Two characters | 2^nd^ spectral moment | 0.53 | 0.51 | 0.79 | 0.24 | 0.53 |
|  |  | Typing speed | Two characters | 3^rd^ spectral moment | 0.54 | 0.49 | 0.78 | 0.24 | 0.53 |
|  |  | Typing speed | Consecutive typing events | Mean log | 0.56 | 0.48 | 0.78 | 0.24 | 0.54 |
|  |  | Typing speed | Consecutive typing events | 50^th^ percentile | 0.55 | 0.45 | 0.77 | 0.23 | 0.52 |
|  |  | Typing speed | Consecutive typing events | 80^th^ percentile | 0.54 | 0.47 | 0.78 | 0.23 | 0.53 |
|  |  | Typing speed | Consecutive typing events | Total (sum) power | 0.56 | 0.48 | 0.79 | 0.25 | 0.54 |
|  |  | Typing speed | Consecutive typing events | Median frequency | 0.56 | 0.4 | 0.76 | 0.21 | 0.52 |
|  |  | Typing speed | Consecutive typing events | Mean frequency | 0.54 | 0.43 | 0.76 | 0.22 | 0.51 |
|  |  | Typing speed | Consecutive typing events | 1^st^ spectral moment | 0.57 | 0.48 | 0.79 | 0.25 | 0.55 |
|  |  | Typing speed | Consecutive typing events | 2^nd^ spectral moment | 0.57 | 0.47 | 0.79 | 0.24 | 0.55 |
|  |  | Typing speed | Consecutive typing events | 3^rd^ spectral moment | 0.57 | 0.46 | 0.78 | 0.24 | 0.54 |
|  |  | Typing speed | ≤ 3 characters in a row | Total (sum) power | 0.56 | 0.49 | 0.79 | 0.25 | 0.54 |
|  |  | Typing speed | ≤ 3 characters in a row | Mean frequency | 0.54 | 0.49 | 0.78 | 0.24 | 0.53 |
|  |  | Typing speed | ≤ 3 characters in a row | 1^st^ spectral moment | 0.56 | 0.50 | 0.79 | 0.25 | 0.54 |
|  |  | Typing speed | ≤ 3 characters in a row | 2^nd^ spectral moment | 0.55 | 0.50 | 0.79 | 0.25 | 0.54 |
|  |  | Typing speed | ≤ 3 characters in a row | 3^rd^ spectral moment | 0.55 | 0.50 | 0.79 | 0.25 | 0.54 |
|  |  | Typing speed | > 3 characters in a row | Mean log | 0.55 | 0.54 | 0.80 | 0.26 | 0.55 |
|  |  | Typing speed | > 3 characters in a row | 10^th^ percentile | 0.58 | 0.54 | 0.81 | 0.28 | 0.57 |
|  |  | Typing speed | > 3 characters in a row | 20^th^ percentile | 0.57 | 0.50 | 0.80 | 0.26 | 0.56 |
|  |  | Typing speed | > 3 characters in a row | 50^th^ percentile | 0.53 | 0.54 | 0.80 | 0.25 | 0.53 |
|  |  | Typing speed | > 3 characters in a row | 80^th^ percentile | 0.54 | 0.50 | 0.78 | 0.24 | 0.53 |
|  |  | Typing speed | > 3 characters in a row | 90^th^ percentile | 0.54 | 0.47 | 0.78 | 0.23 | 0.53 |
|  |  | Typing speed | > 3 characters in a row | Total (sum) power | 0.54 | 0.53 | 0.80 | 0.26 | 0.54 |
|  |  | Typing speed | > 3 characters in a row | Median frequency | 0.54 | 0.47 | 0.78 | 0.24 | 0.53 |
|  |  | Typing speed | > 3 characters in a row | Mean frequency | 0.57 | 0.52 | 0.80 | 0.26 | 0.55 |
|  |  | Typing speed | > 3 characters in a row | 1^st^ spectral moment | 0.54 | 0.54 | 0.80 | 0.26 | 0.54 |
|  |  | Typing speed | > 3 characters in a row | 2^nd^ spectral moment | 0.55 | 0.54 | 0.80 | 0.26 | 0.55 |
|  |  | Typing speed | > 3 characters in a row | 3^rd^ spectral moment | 0.55 | 0.57 | 0.81 | 0.27 | 0.56 |
|  |  | Composite biomarker | | | 0.52 | 0.56 | 0.8 | 0.26 | 0.53 |
| Difficulty with concentration/thinking/fatigue | 413 (72%) | Scroll | Speed of scroll to item | 50^th^ percentile | 0.50 | 0.54 | 0.74 | 0.30 | 0.51 |
|  |  | Scroll | Speed of scroll to item | Median frequency | 0.50 | 0.56 | 0.75 | 0.30 | 0.52 |
|  |  | Scroll | Speed of scroll to item | Mean frequency | 0.51 | 0.53 | 0.74 | 0.29 | 0.51 |
|  |  | Scroll | Speed of scroll to item | 3^rd^ spectral moment | 0.50 | 0.52 | 0.73 | 0.28 | 0.51 |
|  |  | Typing speed | > 3 characters in a row | Mean frequency | 0.56 | 0.49 | 0.74 | 0.30 | 0.54 |
|  |  | Typing speed | > 3 characters in a row | 1^st^ spectral moment | 0.54 | 0.51 | 0.74 | 0.30 | 0.53 |
|  |  | Typing speed | > 3 characters in a row | 2^nd^ spectral moment | 0.55 | 0.53 | 0.75 | 0.31 | 0.54 |
|  |  | Typing speed | > 3 characters in a row | 3^rd^ spectral moment | 0.55 | 0.54 | 0.76 | 0.32 | 0.55 |
|  |  | Composite biomarker | | | 0.48 | 0.61 | 0.73 | 0.34 | 0.52 |

| **Supplemental Table 8.**  Correlation of main adverse posttraumatic neuropsychiatric outcomes after trauma exposure at different timepoints with completion rate of four main assessments (to explore the impact of pre- and post-traumatic symptoms on missing data. | | | | |
| --- | --- | --- | --- | --- |
|  | **Survey** | **Verily Watch** | **Neurocognitive Tests** | **Flash Survey** |
| Pre-Trauma Pain | .0169 | 0.037 | 0.011 | 0.011 |
| Pre-Trauma Depression | -.0167 | -0.007 | -0.021 | -0.011 |
| Pre-Trauma Post-Traumatic Stress Disorder | -.0194 | -0.031 | -0.069^a^ | -0.051^b^ |
| Pre-Trauma Somatic | -.0284 | -0.014 | -0.020 | -0.022 |
| Peritraumatic Pain | -.0131 | -0.052 | -0.020 | -0.027 |
| Peritraumatic Somatic | -.0263 | -0.033 | -0.011 | -0.029 |
| Week 2 Pain | .051^*^ | .062^b^ | 0.032 | 0.046^b^ |
| Week 2 Depression | .0095 | -0.009 | 0.012 | 0.017 |
| Week 2 Post-Traumatic Stress Disorder | .0266 | 0.003 | 0.016 | 0.014 |
| Week 2 Somatic | -.0019 | 0.003 | -0.010 | -0.030 |
| Week 8 Pain | .0211 | 0.070^a^ | 0.014 | 0.054^b^ |
| Week 8 Depression | -.0010 | -0.006 | 0.016 | 0.016 |
| Week 8 Post-Traumatic Stress Disorder | .0059 | 0.003 | 0.021 | 0.024 |
| Week 8 Somatic | -.0032 | 0.035 | 0.005 | 0.006 |
| *Note.* Completion rate was defined as the proportion of finished tasks over the number of the tasks had become available to the participant.  ^a^ Correlation is significant at the 0.01 level (2-tailed) | | | |  |
| ^b^ Correlation is significant at the 0.05 level (2-tailed) | | | |  |

**Supplemental Figure 1.**

Data flow pathways for self-report and Keystroke data collected from trauma survivors participating in an observational cohort study.

Emergency Department Enrollment

Install smartphone App

Bivariate Linear Mixed Model; Bonferroni adjustment for multiple tests

Bivariate Linear Mixed Model; FDR adjustment for multiple tests

Randomly divided into two equal parts for biomarker identification and validation

Selected top 50 variables with the highest absolute correlation that were statistically significant

Keystroke features are merged with self-report symptoms and then within and between subject correlations of keystroke features with each of the 10 APNS constructs were calculated

Self-report Symptoms (Construct Scores)

Keystroke features

Validation Cohort

Identification Cohort

**Final Biomarkers**

Candidate Biomarkers

Signal Processing

Joint Measurement Model for each symptom

Smartphone-based Survey

At 11 timepoints within the first 6 Months

Keystroke behavior data

**
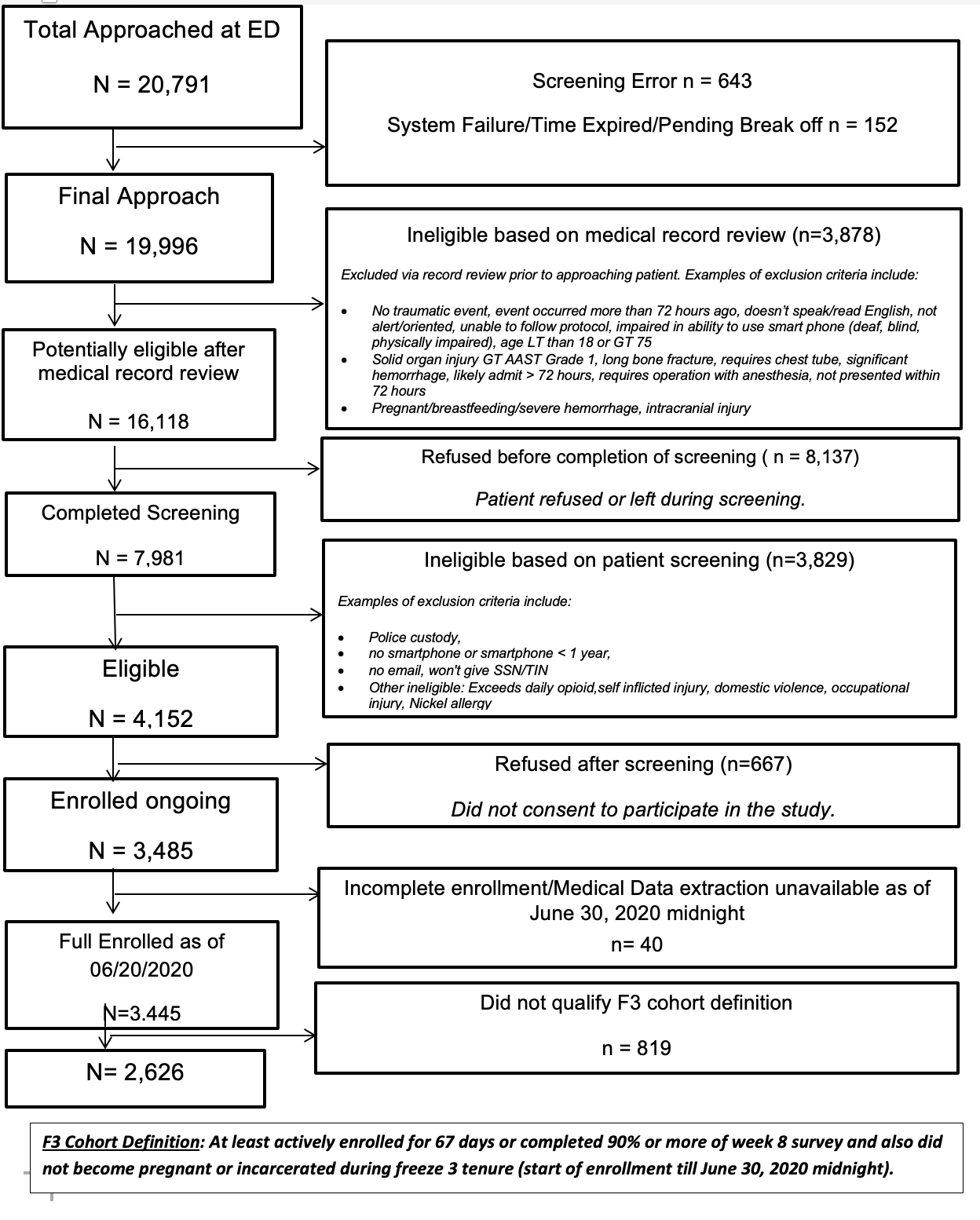
**

**Supplemental Figure 2.**

Visual depiction of study flow and missing data from trauma survivors participating in an observational cohort study.

**Sample Analysis Code.**

**R example code for Data processing.**

library(tidyverse)

library(readxl)

# Load Keystroke features

ks_wide_ANDROI<-read_csv("AURORA_keystroke_imputed_ANDROID.csv") %>% mutate(OS_type="ANDROID")

ks_wide_IOS <- read_csv("AURORA_keystroke_imputed_IOS.csv") %>% mutate(OS_type="IOS")

ks_data <- bind_rows(ks_wide_ANDROID, ks_wide_IOS) %>% select(PID, day, OS_type, everything())

# drop days changed OS_type within 203 days (n=46)

ks_count <- ks_data %>% filter(day<=203) %>% group_by(PID, OS_type) %>% count() %>% ungroup()

PID_switch_OS <- ks_count %>% select(-n) %>% group_by(PID) %>% count() %>% filter(n>1)

ks_data <- ks_data %>% filter(! PID %in% PID_switch_OS$PID)

save(ks_data, file = "AURORA_keystroke_wide.RData")

# Load symptom data and merge with activity features --------------------------

load("AURORA_RDoC_Score_FS_6m_Frz3_Data.RData")

data.aggregation <- function(data, days, agg.var,n.day.before=2, n.day.after=2){

pid <- unique(data$PID)

n.sub <- length(pid)

data.agg <- c()

for(i in 1:n.sub){

data.i <- data[data$PID==pid[i],]

data.i <- data.i[!is.na(data.i$Day),]

OS_Type <- data.i$OS_type %>% max(.,na.rm = T)

data.agg.i <- c()

for(j in days){

temp <- data.i[data.i$Day>=(j-n.day.before) & data.i$Day<=(j+n.day.after),]

agg.i.j <- c(pid[i],j,OS_Type,apply(temp[,agg.var],2,mean,na.rm=TRUE))

data.agg.i <- rbind(data.agg.i, agg.i.j)

}

# data.agg.i <- na.omit(data.agg.i)

data.agg.i <- data.frame(data.agg.i) %>% mutate_at(agg.var, as.numeric)

data.agg.i <- data.agg.i[which(is.na(data.agg.i[,4])==F),] # remove records with missing outcome

data.agg.i <- data.agg.i[which(rowSums(!is.na(data.agg.i[,-(1:4)]))>0),] # remove records with missing ks data

data.agg <- rbind(data.agg, data.agg.i)

}

colnames(data.agg) <- c("PID","day","OS_type","outcome", agg.var[-1])

data.agg <- as.data.frame(data.agg)

return(data.agg)

}

# merge Keystroke and symptom data

mergeDat <- list()

merge.all=TRUE

mergeDat[["pain"]] <- merge(pain.con.score.long,ks_data,by.x=c("PID","Day"),by.y=c("PID","day"),all=merge.all)

mergeDat[["loss"]] <- merge(loss.con.score.long,ks_data,by.x=c("PID","Day"),by.y=c("PID","day"),all=merge.all)

mergeDat[["sleep"]] <- merge(sleep.con.score.long,ks_data,by.x=c("PID","Day"),by.y=c("PID","day"),all=merge.all)

mergeDat[["nightmare"]] <- merge(nightmare.con.score.long,ks_data,by.x=c("PID","Day"),by.y=c("PID","day"),all=merge.all)

mergeDat[["avoidance"]] <- merge(avoid.con.score.long,ks_data,by.x=c("PID","Day"),by.y=c("PID","day"),all=merge.all)

mergeDat[["reexperience"]] <- merge(reex.con.score.long,ks_data,by.x=c("PID","Day"),by.y=c("PID","day"),all=merge.all)

mergeDat[["anxious"]] <- merge(anxious.con.score.long,ks_data,by.x=c("PID","Day"),by.y=c("PID","day"),all=merge.all)

mergeDat[["hyper_arousal"]] <- merge(hyperarousal.con.score.long,ks_data,by.x=c("PID","Day"),by.y=c("PID","day"),all=merge.all)

mergeDat[["somatic_symptom"]] <- merge(somatic.con.score.long,ks_data,by.x=c("PID","Day"),by.y=c("PID","day"),all=merge.all)

mergeDat[["MenFa"]] <- merge(mentalfatigue.con.score.long,ks_data,by.x=c("PID","Day"),by.y=c("PID","day"),all=merge.all)

## aggragate data around the flash survey days

agg.data <- list()

# note that flash survey days are different for different latent constructs, so the following needs

# to be updated accordingly.

fs.pain.days <- unique(pain.con.score.long$Day)

fs.loss.days <- unique(loss.con.score.long$Day)

fs.sleep.days <- unique(sleep.con.score.long$Day)

fs.nightmare.days <- unique(nightmare.con.score.long$Day)

fs.avoid.days <- unique(avoid.con.score.long$Day)

fs.reexp.days <- unique(reex.con.score.long$Day)

fs.anxious.days <- unique(anxious.con.score.long$Day)

fs.arousal.days <- unique(hyperarousal.con.score.long$Day)

fs.somatic.days <- unique(somatic.con.score.long$Day)

fs.MenFa.days <- unique(mentalfatigue.con.score.long$Day)

# a list of the variables that need to be aggregated.

bio.var <- names(ks_data)[-1:-3]

agg.data[["pain"]] <- data.aggregation(mergeDat$pain, fs.pain.days, c("Pain", bio.var), 2, 0)

agg.data[["loss"]] <- data.aggregation(mergeDat$loss, fs.loss.days, c("Loss", bio.var), 2, 0)

agg.data[["sleep"]] <- data.aggregation(mergeDat$sleep, fs.sleep.days, c("Sleep", bio.var), 2, 0)

agg.data[["nightmare"]] <- data.aggregation(mergeDat$nightmare, fs.nightmare.days, c("Nightmare", bio.var), 2, 0)

agg.data[["avoidance"]] <- data.aggregation(mergeDat$avoidance, fs.avoid.days, c("Avoidance", bio.var), 2, 0)

agg.data[["reexperience"]] <- data.aggregation(mergeDat$reexperience, fs.reexp.days, c("Reexperience", bio.var), 2, 0)

agg.data[["anxious"]] <- data.aggregation(mergeDat$anxious, fs.anxious.days, c("Anxious", bio.var), 2, 0)

agg.data[["hyper_arousal"]] <- data.aggregation(mergeDat$hyper_arousal, fs.arousal.days, c("Hyperarousal", bio.var), 2, 0)

agg.data[["somatic_symptom"]] <- data.aggregation(mergeDat$somatic_symptom, fs.somatic.days, c("Somatic", bio.var), 2, 0)

agg.data[["MenFa"]] <- data.aggregation(mergeDat$MenFa, fs.MenFa.days, c("MentalFatigue", bio.var), 2, 0)

# separate bt OS_type

agg.IOS <- lapply(agg.data, function(x) filter(x, OS_type=="IOS"))

agg.IOS <- lapply(agg.IOS, function(x) janitor::remove_empty(x, which = "cols"))

agg.ANDROID <- lapply(agg.data, function(x) filter(x, OS_type=="ANDROID"))

agg.ANDROID <- lapply(agg.ANDROID, function(x) janitor::remove_empty(x, which = "cols"))

# export merge and aggragated data

save(agg.IOS, agg.ANDROID, file = "AURORA_agg_keystroke_sep_OS.RData")

**R example code for within and between subject correlations of keystroke features**

library(tidyverse)

library(psych)

correlations_ANDROID <- list()

for (name in names(agg.ANDROID)) {

result <- data.frame()

data <- agg.ANDROID[[name]]

data$PID <- as.numeric(data$PID)

# vars=names(data[-1:-4])

# include vars with more than 60 records available

vars=names(data[-(1:4)])[which(colSums(is.na(data[-(1:4)])==F)>60)]

for (i in vars) {

corrs <- statsBy(data %>% select(PID, outcome, i), group = "PID", cors = T, na.rm = T)

# print(corrs,short=FALSE)

temp <- data.frame(outcome=name,

variable=i,

between.cor=corrs$rbg[2,1] %>% round(4),

between.p.value=corrs$pbg[2,1] %>% round(4),

within.cor=corrs$rwg[2,1] %>% round(4),

within.p.value=corrs$pwg[2,1] %>% round(4))

result <- rbind(result, temp)

print(i)

}

correlations_ANDROID[[name]] <- result

}

**R example code for biomarker identification and validation**

library(tidyverse)

# get vars that with highest absolute value of correlation which adj.p.value <0.05

correlations_ANDROID <- correlations_ANDROID %>%

map( ~ mutate(., adj.btw.p.value=round(p.adjust(between.p.value, method = "fdr"),4),

adj.wi.p.value=round(p.adjust(within.p.value, method = "fdr"),4)))

cor_btw <- correlations_ANDROID %>%

map( ~ filter(., adj.btw.p.value<0.05)) %>%

map( ~ arrange(., desc(abs(between.cor))))

vars_btw <- cor_btw %>%

map(~ slice(.,1:50)) %>%

map(~ select(., c(outcome, variable))) %>%

map(~ mutate(., btw=T))

cor_wi <- correlations_ANDROID %>%

map( ~ filter(., adj.wi.p.value<0.05)) %>%

map( ~ arrange(., desc(abs(within.cor))))

vars_wi <- cor_wi %>%

map(~ slice(.,1:50)) %>%

map(~ select(., c(outcome, variable))) %>%

map(~ mutate(., wi=T))

cor_vars <- Map(bind_rows, vars_btw, vars_wi) %>% map(~ select(., c(outcome, variable))) %>% map(~ distinct(.))

# split test and valid data -----------------------------------------------

all.var <- gsub(paste("pain",".",sep = ""), "",colnames(as.data.frame(agg.ANDROID["pain"])))

var.list <- all.var[-1:-4]

data <- agg.ANDROID %>% map_df(select, c(all.var)) %>% arrange(PID, day) %>% select(-outcome) %>% distinct()

n <- data$PID %>% unique() %>% length()

set.seed(1234)

test.PID <- sample(data$PID %>% unique(), size = n*0.5)

data.test <- data %>% filter(PID %in% test.PID)

data.valid <- data %>% filter(!PID %in% test.PID)

agg.test <- lapply(agg.ANDROID, function(x) filter(x, PID %in% test.PID))

agg.valid <- lapply(agg.ANDROID, function(x) filter(x, !PID %in% test.PID))

# standardize

agg.test <- lapply(agg.test, function(x) x %>% mutate_at(var.list, scale))

agg.valid <- lapply(agg.valid, function(x) x %>% mutate_at(var.list, scale))

save(data.test, data.valid, agg.test, agg.valid, file = "Data/split_ANDROID.RData")

# biomarker identification

correlations_ANDROID_test <- list()

for (name in names(agg.test)) {

result <- data.frame()

data <- agg.test[[name]]

vars <- cor_vars[[name]]$variable

for (i in vars) {

data$PID <- as.numeric(data$PID)

corrs <- statsBy(data %>% select(PID, outcome, i), group = "PID", cors = T, na.rm = T)

temp <- data.frame(outcome=name,

variable=i,

between.cor=corrs$rbg[2,1] %>% round(4),

between.p.value=corrs$pbg[2,1],

between.lower=corrs$ci.bg[[1]][1,1] %>% round(4),

between.upper=corrs$ci.bg[[1]][1,3] %>% round(4),

within.cor=corrs$rwg[2,1] %>% round(4),

within.p.value=corrs$pwg[2,1],

within.lower=corrs$ci.wg[[1]][1,1] %>% round(4),

within.upper=corrs$ci.wg[[1]][1,3] %>% round(4))

result <- rbind(result, temp)

print(i)

}

correlations_ANDROID_test[[name]] <- result

}

# biomarker validation

correlations_ANDROID_valid <- list()

start <- Sys.time()

for (name in names(agg.valid)) {

result <- data.frame()

data <- agg.valid[[name]]

vars <- cor_vars[[name]]$variable

for (i in vars) {

data$PID <- as.numeric(data$PID)

corrs <- statsBy(data %>% select(PID, outcome, i), group = "PID", cors = T, na.rm = T)

temp <- data.frame(outcome=name,

variable=i,

between.cor=corrs$rbg[2,1] %>% round(4),

between.p.value=corrs$pbg[2,1],

between.lower=corrs$ci.bg[[1]][1,1] %>% round(4),

between.upper=corrs$ci.bg[[1]][1,3] %>% round(4),

within.cor=corrs$rwg[2,1] %>% round(4),

within.p.value=corrs$pwg[2,1],

within.lower=corrs$ci.wg[[1]][1,1] %>% round(4),

within.upper=corrs$ci.wg[[1]][1,3] %>% round(4))

result <- rbind(result, temp)

print(i)

}

correlations_ANDROID_valid[[name]] <- result

}

1. Todd KH, Funk KG, Funk JP, Bonacci R. Clinical significance of reported changes in pain severity. *Annals of emergency medicine.* 1996;27(4):485-489.

2. Ulirsch J, Ballina L, Soward A, et al. Pain and somatic symptoms are sequelae of sexual assault: results of a prospective longitudinal study. *Eur J Pain.* 2014;18(4):559-566.

3. Cella D, Riley W, Stone A, et al. The Patient-Reported Outcomes Measurement Information System (PROMIS) developed and tested its first wave of adult self-reported health outcome item banks: 2005–2008. *Journal of clinical epidemiology.* 2010;63(11):1179-1194.

4. Monk TH, Buysse DJ, Kennedy KS, Potts JM, DeGrazia JM, Miewald JM. Measuring sleep habits without using a diary: the sleep timing questionnaire. *Sleep.* 2003;26(2):208-212.

5. Blake DD, Weathers FW, Nagy LM, et al. The development of a clinician-administered PTSD scale. *Journal of traumatic stress.* 1995;8(1):75-90.

6. Pilkonis PA, Choi SW, Reise SP, et al. Item banks for measuring emotional distress from the Patient-Reported Outcomes Measurement Information System (PROMIS®): depression, anxiety, and anger. *Assessment.* 2011;18(3):263-283.

7. Weathers FW, Litz BT, Keane TM, Palmieri PA, Marx BP, Schnurr PP. The PTSD checklist for DSM-5 (PCL-5). 2013. ptsd.va.gov.

8. Auvergne L, Bortsov AV, Ulirsch JC, et al. Association of Epidemiologic Factors and Genetic Variants Influencing Hypothalamic-Pituitary-Adrenocortical Axis Function With Postconcussive Symptoms After Minor Motor Vehicle Collision. *Psychosom Med.* 2016;78(1):68-78.
